# May the Phage be With You? Prophage-Like Elements in the Genomes of Soft Rot *Pectobacteriaceae*: *Pectobacterium* spp. and *Dickeya* spp.

**DOI:** 10.3389/fmicb.2019.00138

**Published:** 2019-02-14

**Authors:** Robert Czajkowski

**Affiliations:** Laboratory of Biologically Active Compounds, Intercollegiate Faculty of Biotechnology of University of Gdansk and Medical University of Gdansk, University of Gdansk, Gdansk, Poland

**Keywords:** *Pectobacterium* spp., *Dickeya* spp., integrase, attachment site, holin, lysin, bacterial gene, ecological fitness

## Abstract

Soft Rot *Pectobacteriaceae* (SRP; *Pectobacterium* spp. and *Dickeya* spp., formerly known as pectinolytic *Erwinia* spp.) are necrotrophic bacterial pathogens infecting a large number of plant species worldwide, including agriculturally-important crops. Despite the SRP importance in agriculture, little is known about the bacteriophages infecting them, and even less about the prophages present in their genomes. Prophages are recognized as factors underlying bacterial virulence, genomic diversification and ecological fitness that contribute to the novel phenotypic properties of bacterial hosts. Likewise, they are recognized as a driving force of bacterial evolution. In this study, 57 complete genomes of *Pectobacterium* spp. and *Dickeya* spp. deposited in NCBI GenBank, were analyzed for the presence of prophage-like elements. Viral sequences were discovered in 95% of bacterial genomes analyzed with the use of PHASTER, PhiSpy, and manual curation of the candidate sequences using NCBI BLAST. In total 37 seemingly intact and 48 putatively defective prophages were found. The 37 seemingly intact prophages (27 sequences in *Dickeya* spp. genomes and 10 sequences in *Pectobacterium* spp. genomes) were annotated using RAST. Analysis of the prophage genes encoding viral structural proteins allowed classification of these prophages into different families of the order *Caudovirales* (tailed bacteriophages) with the SRP prophages of the *Myoviridae* family (81% of found prophages) being the most abundant. The phylogenetic relationships between prophages were analyzed using amino acid sequences of terminase large subunit (gene *terL*), integrase (gene *int*), holin (gene *hol*), and lysin (gene *lys*). None of these markers however proved fully useful for clear phylogenetic separation of prophages of SRP into distinct clades. Comparative analyses of prophage proteomes revealed six clusters: five present in *Dickeya* spp. and one within *Pectobacterium* spp. When screened for the presence of bacterial genes in the genomes of intact prophages, only one prophage did not contain any ORFs of bacterial origin, the other prophages contained up to 23 genes acquired from bacterial hosts. The bacterial genes present in prophages could possibly affect fitness and virulence of their hosts. The implication of prophage presence in the genomes of *Pectobacterium* spp. and *Dickeya* spp. is discussed.

## Introduction

It is generally accepted that phages are the most abundant biological entities in the environment with an estimated number of 10^31^ particles on Earth. Consequently, they are present in virtually all habitats in which bacteria exist (Suttle, 2007). Based on their particular relationship with a host, they can be either lytic or temperate (Ackermann, 2003). Temperate bacteriophages integrate their genetic material into the host genome and persist inside bacterial cells as so-called prophages (Weinbauer, 2004). After integration, prophages are maintained in a host cell, undergoing non-lytic growth typically called a lysogenic state (Canchaya et al., 2004). During lysogeny phage DNA remains inactive, except for some regulatory and accessory genes, which are required to maintain the dormant state of the virus. This dormant bacteriophage DNA may constitute up to 20% of the host genome (Casjens, 2003).

The occurrence of prophages can contribute greatly to bacterial fitness (Bondy-Denomy and Davidson, 2014; Nanda et al., 2014). Prophages can influence host variability and evolution and may determine the adaptation of their hosts to specific ecological niches (Wang et al., 2010; Fortier and Sekulovic, 2013; Varani et al., 2013). The presence of prophages may affect bacterial genomes in several ways. For example, their integration is responsible for gene disruption or translocation which, in turn, may confer phenotypic changes in the host. Similarly, prophages may introduce new traits into the host, such as pathogenicity determinants that alter bacterial fitness. These new traits might also modulate the switch between lytic and lysogenic cycles (Brüssow et al., 2004). Consequently, prophages have been studied in a number of bacterial species including plant pathogens to understand their role in bacterial ecology (Casjens, 2003; Varani et al., 2013). To date, however, they have not been extensively studied in the Soft Rot *Pectobacteriaceae* (SRP).

Plant pathogenic Soft Rot *Pectobacteriaceae* (Adeolu et al., 2016) [consisting of *Pectobacterium* spp. and *Dickeya* spp., formerly characterized as pectinolytic *Erwinia* spp. (Pérombelon, 2002)] are considered to be among the top ten most important agricultural phytopathogens (Mansfield et al., 2012). They cause significant losses in crop production (up to 40%) with disease severity dependent on weather conditions, plant susceptibility and pathogen inoculum. Among the economically most important hosts worldwide are potato, carrot, tomato, onion, pineapple, maize, rice, hyacinth, chrysanthemum, and calla lily (Perombelon and Kelman, 1980; Charkowski, 2018). SRP are widespread in various ecological niches including bulk and rhizosphere soils, water, sewage, the surface of host and non-host plants, and the surfaces and interior of insects (Perombelon and Kelman, 1980; Grenier et al., 2006; Rossmann et al., 2018). Because of the diverse habitats in which they can be found, bacteria presumably also exhibit diverse lifestyles because of their transfer between these different environments; for example, from plants to soil, from plant to plant, from host plant to non-host plant, from surface and/or irrigation water to plants, from water to soil, and *vice versa*) (Charkowski, 2018). In all of these surroundings the SRP can encounter lytic and temperate bacteriophages and hence may become easily and repeatedly infected (Canchaya et al., 2004).

The knowledge of prophages present in SRP genomes is currently very limited as only a few temperate bacteriophages that specifically infect *Pectobacterium* spp. and *Dickeya* spp. have been characterized (for review see: Varani et al., 2013; Czajkowski, 2016). The viruses that have been characterized include temperate bacteriophage ΦEC2 infecting *D. dadantii* and *D. solani* (Resibois et al., 1984); bacteriophage ZF40 (Korol and Tovkach, 2012) infecting *P. carotovorum* subsp. *carotovorum;* bacteriophages phiTE (Blower et al., 2012), phiM1 (Blower et al., 2017), ECA29 and ECA41 (Evans et al., 2010) infecting *P. atrosepticum;* and bacteriophages LIMEstone 1 and LIMEstone2 infecting *D. solani* (Adriaenssens et al., 2012; Day et al., 2017). Likewise, the biological role of only two prophages present in SRP genomes (ECA29 and ECA41 localized in the genome of *P. atrosepticum* strain SCRI1043) have been elucidated to date as being involved in modulation of host swimming motility and virulence in potato (Evans et al., 2010).

The aim of this study was to identify prophage-like sequences in the complete genome sequences of *Pectobacterium* spp. and *Dickeya* spp. strains deposited in GenBank (NCBI) and to characterize these prophages using comparative genomic tools. The implications of the presence of prophage in SRP genomes and the way these genetic elements may contribute to ecological fitness of *Pectobacterium* spp. and *Dickeya* spp. are also discussed.

## Materials and Methods

### Data Collection and Identification of Candidate Prophage Sequences in Complete Genomes of *Dickeya* spp. and *Pectobacterium* spp.

**Figure 1:**
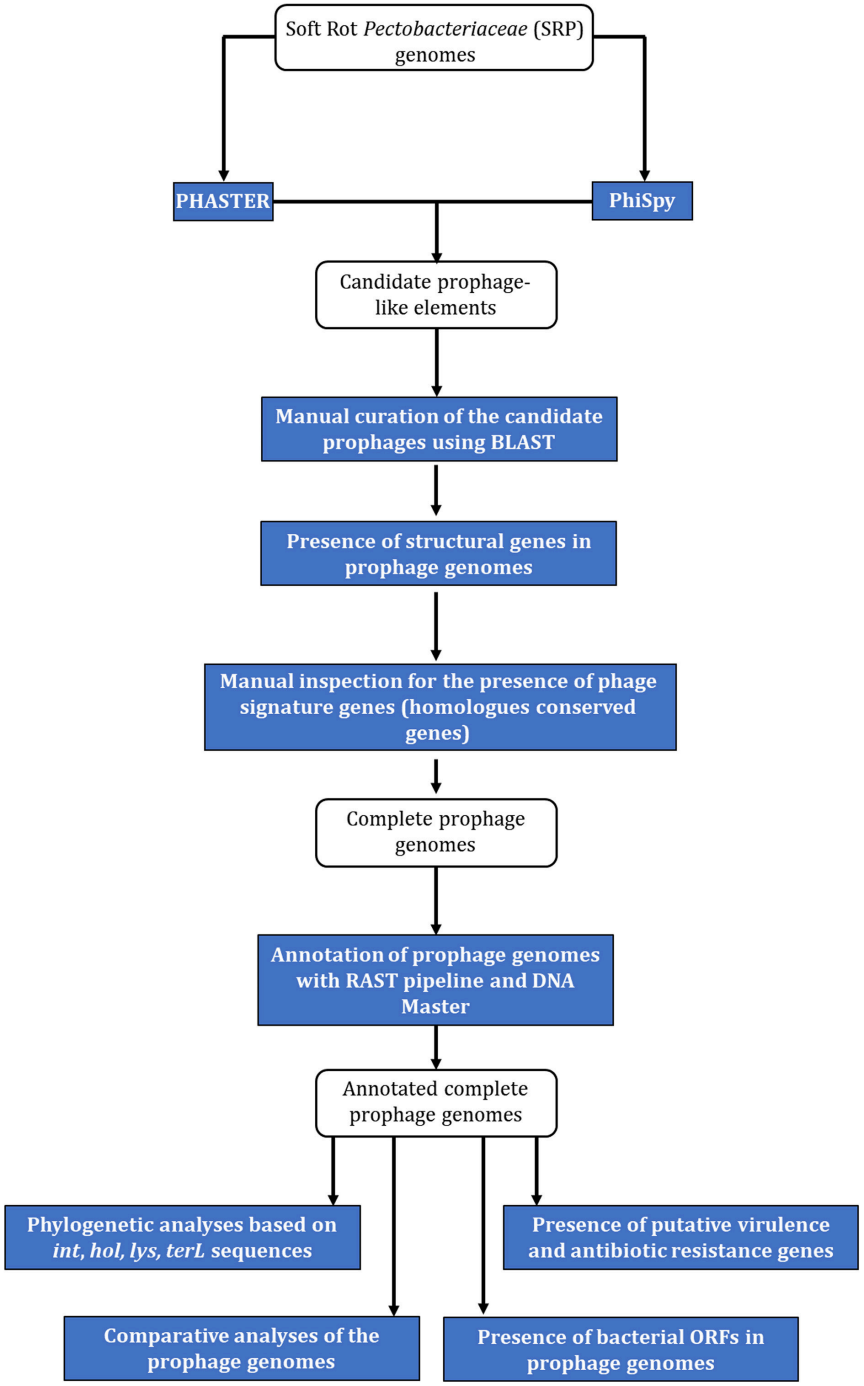
The workflow for identification and characterization of SRP prophages. The blue rectangles represent the tools and methods used for identification of prophages' sequences in bacterial genomes and the white rounded rectangles represent the (input) data used for the analyses.

The strategy used to identify and characterize prophages in SRP genomes is presented in Figure 1. Fifty seven complete genome sequences (17 *Pectobacterium* spp. genomes and 40 *Dickeya* spp. genomes) were accessed from NCBI (National Center for Biotechnology Information, https://www.ncbi.nlm.nih.gov/) (August 2018) (Table 1).

**Table 1:** Genomic features of intact prophages present in the complete genomes of Soft Rot *Pectobacteriaceae* (*Dickeya* spp. and *Pectobacterium* spp.) obtained from GenBank (NCBI).

**No**.	**Prophage**	**Host, (GenBank accession)**	**Coordinates in bacterial genome**	**Prophage size (kb)**	**Putative phage attachment region**	**Classification according to VirFam (Lopes et al., 2014)**	**The phage with the highest number of proteins most similar ^a^ to those found in the prophage (GenBank accession, no. similar proteins)**	**ORFs in the host genome flanking the integrated prophage sequence, L: left flank, R: right flank**
**PROPHAGES PRESENT IN GENOMES OF** ***DICKEYA*** **SPP**.								
1	phiDda1	*Dickeya dadantii* 3937, (NC_014500.1)	915,372 – 947,293	31.9	GGGAGTTGAACCCGCGTCCGAAATTCCTACA	*Myoviridae*	*Haemophilus* phage HP1 (NC_001697, 17)	L: Hcp family type VI secretion system effector R: ssrA – transfer-messenger RNA
2	phiDda3	*Dickeya dadantii* DSM 18020, (NZ_CP023467.1)	4,707,016 – 4,750,917	43.9	AAATTGATAAGA	*Myoviridae*	*Salmonella* phage SEN5 (NC_028701, 22)	L: N-carbamoylputrescine amidase R: stress resistance protein
3	phiDda4	*Dickeya dadantii* NCPPB 898, (NZ_CM001976.1)	174,591 – 209,384	34.7	ACACCATCCCTGTCTTTCGCCCTCCTTGATGGAGGGCTTTTTTTTG	*Myoviridae*	*Salmonella* phage SEN5 (NC_028701, 22)	L: stress resistance protein R: transposase
4	phiDdd1	*Dickeya dadantii* subsp. *dieffenbachiae* NCPPB 2976, (NZ_CM001978.1)	861,130 – 909,093	47.9	TATTACGTTGAAA	*Myoviridae*	*Haemophilus* phage HP1 (NC_001697, 17)	L: nucleotidyl transferase AbiEii/AbiGii toxin family protein R: TonB-family protein
5	phiDdd2	*Dickeya dadantii* subsp. *dieffenbachiae* NCPPB 2976, (NZ_CM001978.1)	2,975,246 – 3,010,542	35.2	ATCAAGGCGTTAGCACATGGTGCCCAGAGCGGGACTTGAACCCGCACAGCGCGAACGCCGAGGGATTTTAAA	*Myoviridae*	*Enterobacteria* phage P88 (NC_026014, 21)	L: pectate lyase R: tRNA-Leu
6	phiDda6	*Dickeya dadantii* NCPPB 3537, (NZ_CM001982.1)	863,862 – 896,397	32.5	TGGTGGAGCTGGGGGGAGTTGAACCCCCGTCCGAAATTCCTACA	*Myoviridae*	*Haemophilus* phage HP1 (NC_001697, 17)	L: hypothetical protein R: type II toxin-antitoxin system RatA family toxin
7	phiDdi1	*Dickeya dianthicola* RNS04.9, (NZ_CP017638.1)	361,591 – 422,414	60.8	GGGTTTTTTGTT	*Myoviridae*	*Enterobacteria* phage Fels-2 (NC_010463, 28)	L: TetR/AcrR family transcriptional regulator R: transcription termination/antitermination protein NusA
8	phiDdi3	*Dickeya dianthicola* NCPPB 453, (NZ_CM001841.1)	599,893 – 660,716	60.8	GGGTTTTTTGTT	*Myoviridae*	*Enterobacteria* phage Fels-2 (NC_010463, 28)	L: TetR/AcrR family transcriptional regulator R: ribosome maturation factor RimP
9	phiDdi5	*Dickeya dianthicola* GBBC 2039, (NZ_CM001838.1)	1,003,276 – 1,070,503	67.2	TGCTGAAAAGTG	*Myoviridae*	*Haemophilus* phage HP2 (NC_003315, 17)	L: MATE family efflux transporter R: GNAT family acetyltransferase
10	phiDdi6	*Dickeya dianthicola* NCPPB 3534, (NZ_CM001840.1)	794,198 – 850,479	56.2	TGCTGAAAAGTG	*Myoviridae*	*Haemophilus* phage HP2 (NC_003315, 17)	L: MATE family efflux transporter R: GNAT family acetyltransferase
11	phiD2	*Dickeya* sp. CSL RW240, (NZ_CM001973.2)	2,423,239 – 2,497,674	74.4	ATGTTTTTAATC	*Myoviridae*	*Salmonella* phage SEN5 (NC_028701, 22)	L: hemagglutinin R: hypothetical protein
12	phiD3	*Dickeya* sp. NCPPB 3274, (NZ_CM001979.1)	842,418 – 875,475	33	CGTCCGAAATTCCTACA	*Myoviridae*	*Haemophilus* phage HP2 (NC_003315, 16)	L: hypothetical protein R: type II toxin-antitoxin system RatA family toxin
13	phiD4	*Dickeya* sp. NCPPB 3274, (NZ_CM001979.1)	3,154,528 – 3,194,907	40.3	TCTTATATTACAAGGCGTTAGCACATGGTGCCCAGAGCGGGACTTGAACCCGCACAGCGCGAACGCCGAGGGATTTTAAA	*Myoviridae*	*Enterobacteria* phage P88 (NC_026014, 21)	L: pectate lyase R: two-component system response regulator UvrY
14	phiD5	*Dickeya* sp. NCPPB 569, (NZ_CM001975.1)	2,632,447 – 2,670,943	38.4	TTATATCACAAGCACTTATCGCATGGTGCCCAGAGCGGGACTTGAACCCGCACAGCGCGAACGCCGAGGGATTTTAAA	*Myoviridae*	*Enterobacteria* phage P88 (NC_026014, 21)	L: methyl-accepting chemotaxis protein R: two-component system response regulator UvrY
15	phiD6	*Dickeya* sp. NCPPB 569, (NZ_CM001975.1)	2,672,697 – 2,736,528	63.8	TCAATTAATTTC	*Myoviridae*	*Edwardsiella* phage GF-2 (NC_026611, 20)	L: two-component system response regulator UvrY R: hypothetical protein
16	phiDze1	*Dickeya zeae* Ech586, (NC_013592.1)	818,289 – 848,156	29.8	TGGTGGAGCTGGGGGGAGTTGAACCCCCGTCCGAAATTCCTACA	*Myoviridae*	*Haemophilus* phage HP1 (NC_001697, 18)	L: acyltransferase R: SsrA-binding protein SmpB
17	phiDze2	*Dickeya zeae* EC1, (NZ_CP006929.1)	2,784,148 – 2,821,964	37.8	GCATGGTGCCCAGAGCGGGACTTGAACCCGCACAGCGCGAACGCCGAGGGATTTTAAA	*Myoviridae*	*Enterobacteria* phage P88 (NC_026014, 21)	L: pectate lyase R: two-component system response regulator UvrY
18	phiDze3	*Dickeya zeae* NCPPB 3532, (NZ_CM001858.1)	3,123,456 – 3,170,215	46.7	TGGCGGAGGAGTAGAGATTCGAACTCTAGAACGCTTTCGCGTCGCCGGTTTTCAAGACCGG	*Myoviridae*	*Enterobacteria* phage P88 (NC_026014, 16)	L: isochorismatase family protein R: acylphosphatase
19	phiDze4	*Dickeya zeae* CSL RW192, (NZ_CM001972.1)	2,512,118 – 2,570,347	58.2	CTGGCGTTACCGGT	*Myoviridae*	*Enterobacteria* phage P88 (NC_026014, 24)	L: histidinol dehydrogenase R: Tat proofreading chaperone DmsD
20	phiDze5	*Dickeya zeae* NCPPB 3531, (NZ_CM001980.1)	2,400,063 – 2,457,314	57.2	CTGGCGTTACCGGT	*Myoviridae*	*Enterobacteria* phage P88 (NC_026014, 25)	L: histidinol dehydrogenase R: Tat proofreading chaperone DmsD
21	phiDze6	*Dickeya zeae* MK19, (NZ_CM001985.1)	1,700,269 – 1,739,933	39.6	TTTAAAATCCCTCGGCGTTCGCGCTGTGCGGGTTCAAGTCCC	*Myoviridae*	*Enterobacteria* phage P88 (NC_026014, 21)	L: two-component system response regulator UvrY R: pectate lyase
22	phiDda2	*Dickeya dadantii* DSM 18020, (NZ_CP023467.1)	1,947,566 – 2,002,033	54.4	CCGGTCTCGAAAACCGGAGTAGGGGCAACTCTACCGGGGGTTCAAATCCCCCTCTCTCC	*Siphoviridae*	*Pectobacterium* phage ZF40 (NC_019522, 19)	L: type IV secretion protein Rhs R: molybdopterin-synthase adenylyltransferase MoeB
23	phiDpa1	*Dickeya paradisiaca* Ech703, (CP001654)	4,420,522 – 4,456,935	36.4	TGTGGTTAATGA	*Siphoviridae*	*Salmonella* phage SEN5 (NC_028701, 18)	L: fructose-1,6-bisphosphatase, class II R: putative stress resistance protein
24	phiDpa2	*Dickeya paradisiaca* NCPPB 2511, (NZ_CM001857.1)	4,377,338 – 4,413,753	36.4	TGTGGTTAATGA	*Siphoviridae*	*Salmonella* phage SEN5 (NC_028701, 18)	L: class II fructose-bisphosphatase R: envelope stress sensor histidine kinase CpxA
25	phiDso1	*Dickeya solani* ND14b, (NZ_CP009460.1)	4,559,121 – 4,616,737	57.6	GGATTAACAGTC	*Siphoviridae*	*Pectobacterium* phage ZF40 (NC_019522, 20)	L: XRE family transcriptional regulator R: pectate lyase
26	phiD1	*Dickeya* sp. CSL RW240, (NZ_CM001973.2)	1,717,167 – 1,770,740	53.5	GATTGATTTTCTGACATTT	*Podoviridae*	*Enterobacteria* phage 933W (NC_000924, 14)	L: tRNA 2-thiocytidine synthetase TtcA R: hypothetical protein
27	phiDch1	*Dickeya chrysanthemi* NCPPB 516, (NZ_CM001904.1)	1,598,056 – 1,638,180	40.1	TTTAAAATCCCTCGGCGTTCGCGCTGTGCGGGTTCAAGTCCC	*Podoviridae*	*Enterobacteria* phage Fels-2 (NC_010463, 26)	L: two-component system response regulator UvrY R: pectate lyase
**PROPHAGES PRESENT IN GENOMES OF** ***PECTOBACTERIUM*** **SPP**.								
28	phiPcc1	*Pectobacterium carotovorum* subsp. *carotovorum* PC1, (NC_012917.1)	2,986,224 – 3,022,684	36.4	TTAAATCAATGGTGCCCGGGGCGGGACTTGAACCCGCACAGCGCGAACGCCGAGGGATTTTAAA	*Myoviridae*	*Salmonella* phage SEN4 (NC_029015, 21)	L: methyl-accepting chemotaxis protein R: CDP-diacylglycerol-glycerol-3-phosphate 3-phosphatidyltransferase
29	phiPcc2	*Pectobacterium carotovorum* subsp. *carotovorum* PCC21, (NC_018525.1)	2,088,340 – 2,136,029	47.6	TTCGGTCTTTTTTTT	*Myoviridae*	*Salmonella* phage SEN34 (NC_028699, 28)	L: formate-dependent phosphoribosylglycinamide formyltransferase R: hypothetical protein
30	phiPc1	*Pectobacterium carotovorum* subsp. *odoriferum* BC S7, (CP009678.1)	2,103,457 – 2,155,798	52.3	TCGGTCTTTTTTTT	*Myoviridae*	*Pectobacterium* phage ZF40 (NC_019522, 30)	L:phosphoribosylglycinamide formyltransferase R: hypothetical protein
31	phiPc2	*Pectobacterium carotovorum* subsp. *odoriferum* BC S7, (CP009678.1)	2,995,809 – 3,034,426	38.6	ATCAATGGTGCCCGGGGCGGGACTTGAACCCGCACAGCGCGAACGCCGAGGGATTTTAAA	*Myoviridae*	*Salmonella* phage SEN34 (NC_028699, 28)	L: helicase R: tRNA-Cys
32	phiPa1	*Pectobacterium atrosepticum* JG10-08, (NZ_CP007744.1)	2,844,474 – 2,922,530	78	AACAAATAGCCA	*Myoviridae*	*Haemophilus* phage HP1 (NC_001697, 16)	L: amidohydrolase R: thioredoxin-disulfide reductase
33	phiPa2	*Pectobacterium atrosepticum* 21A, (NZ_CP009125.1)	2,122,794 – 2,157,360	34.5	ACCGATTACTGAC	*Myoviridae*	*Haemophilus* phage HP1 (NC_001697, 16)	L:hydantoinase/ oxoprolinase family protein R: formate transporter FocA
34	phiPa3	*Pectobacterium atrosepticum* 21A, (NZ_CP009125.1)	4,721,943 – 4,754,520	32.5	AAAAAAAAGCCCTCCATCATGGAGGGC	*Myoviridae*	*Salmonella* phage SEN5 (NC_028701, 25)	L: sugar transporter R: periplasmic heavy metal sensor
35	phiPpa1	*Pectobacterium parmentieri* RNS 08-42-1A, (NZ_CP015749.1)	2,265,306 – 2,337,688	72.3	TATTAAAATAAT	*Myoviridae*	*Enterobacteria* phage fiAA91-ss (NC_022750, 15)	L: DNA-binding protein R: relaxase
36	phiPwa2	*Pectobacterium wasabiae* CFBP 3304, (NZ_CP015750.1)	3,916,413 – 3,968,304	51.8	ACGATAAAAATC	*Myoviridae*	*Enterobacteria* phage PsP3 (NC_005340, 18)	L: zinc ABC transporter ATP-binding protein ZnuC R: calcium-binding protein
37	phiPwa1	*Pectobacterium wasabiae* CFBP 3304, (NZ_CP015750.1)	3,624,405 – 3,685,611	61.2	GCAGACGGTGAA	*Siphoviridae*	*Enterobacteria* phage SfV (NC_003444, 12)	L: glutamine ABC transporter substrate-binding protein GlnH R: Bcr/CflA family multidrug efflux MFS transporter

a *At least 70% amino acid identity over the whole protein length*.

Candidate prophage-like elements were identified with PHASTER (http://phaster.ca/) using settings described in (Arndt et al., 2016) and with PhiSpy (https://edwards.sdsu.edu/PhiSpy/index.php) using settings described in Akhter et al. (2012), followed by manual inspection of the sequences for the presence of signature genes: attachment sites (*att*), gene(s) encoding integrase(s), terminases(s), transposases(s), genes coding for structural viral proteins and the sequences of prophage integration sites, as suggested by others (Boyd and Brüssow, 2002). The candidate prophage-like element was defined as seemingly intact prophage when its sequence contained altogether: (i) phage attachment sites, (ii) genes encoding structural phage proteins, (iii) genes coding for proteins involved in DNA regulation, insertion to the host genome and lysis. Consequently, the candidate prophage-like element was defined as putatively defective when its sequence lacks one or more features (genes) as described above (Akhter et al., 2012; Arndt et al., 2016).

Furthermore, any two seemingly intact prophages were characterized as the same prophage if their genomes shared at least 95% nucleotide identity. Prophages were characterized on the basis of their homology with known phage sequences deposited in NCBI GenBank (https://www.ncbi.nlm.nih.gov/genbank/) using NCBI BLAST (https://blast.ncbi.nlm.nih.gov/Blast.cgi). The presence of structural genes in prophage sequences was verified by the VirFam (http://biodev.cea.fr/virfam/) using settings described in Lopes et al. (2014).

### Analyses of Prophage Genome Sequences and Comparative Genomics

Prophage sequences were annotated using RAST (Rapid Annotation using Subsystem Technology) (rast.nmpdr.org) as described in Aziz et al. (2008), Brettin et al. (2015) (computational settings: Classic RAST, Glimmer3 release 70, domain Viruses, genetic code:11, disable replication), and DNA Master (Lawrence, University of Pittsburgh, Pennsylvania, USA) (http://en.bio-soft.net/dna/dnamaster.html) using settings advised in Pope and Jacobs-Sera (2018).

The *attL* and *attR* attachment sites were identified using PHASTER (http://phaster.ca/) as described in Arndt et al. (2016) and manually inspected using CLC Main Workbench 7 (Qiagen) by assessing the phage localization in the host genome. Multiple sequence alignment of individual prophage genes and phylogenetic analyses were performed using Phylogenetic Pipeline of Information Génomique et Structurale, CNRS-AMU, France (http://www.phylogeny.fr/).

Because of the lack of a universal genetic marker in bacteriophages (Lawrence et al., 2002; Adriaenssens and Cowan, 2014), phylogenetic characterization of bacterial viruses and prophages may be based on comparison of different sequences e.g., encoding integrase, large subunit of terminase, holin and/or lysin (syn. endolysin, murein hydrolase). Amino acid sequences derived from *int, hol, lys, terL*, respectively, were used to phylogenetically analyze the 37 seemingly intact prophages in this study. For this, sequences were aligned with MUSCLE (v3.8.31) configured for highest accuracy (MUSCLE with default settings), after alignment, ambiguous regions (i.e., containing gaps and/or poorly aligned) were removed with Gblocks (v0.91b) using the following parameters: (i) minimum length of a block after gap cleaning equal to 10, (ii) no gap positions were allowed in the final alignment, (iii) all segments with contiguous non-conserved positions bigger than 8 were rejected, (iv) minimum number of sequences for a flank position equal to 85%, graphical representation and edition of the phylogenetic tree were performed with TreeDyn (v198.3).

Comparative analyses of the prophage genomes were done using EDGAR (Blom et al., 2009) accessed via (https://edgar.computational.bio.uni-giessen.de) with settings described in Blom et al. (2009), DNA Master (Lawrence, University of Pittsburgh, Pennsylvania, USA) (http://en.bio-soft.net/dna/dnamaster.html) and BLASTn (accessed via (https://blast.ncbi.nlm.nih.gov/Blast.cgi). Pairwise comparison of sequences (BLAST) (Altschul et al., 1990) was analyzed using MAUVE as described in Darling et al. (2010) (computational settings: alignment with progressiveMauve (aligner: Muscle 3.6), default seed weight (15), full alignment (minimum island size: 50, maximum backbone gap size: 50, minimum backbone size: 50), use of seed families: yes, iterative refinement: yes, determination of LCBs: yes), and DNA Master.

The presence of genes of bacterial origin in the prophage genomes was assessed by NCBI BLAST searches. For this, the gene was classified as being of bacterial origin when altogether: (i) the gene (encoding known or hypothetical protein) is frequently present in bacterial genome(s), (ii) is unnecessary to complete bacteriophage life cycle, (iii) encodes protein with an enzymatic activity not required by the virus to interact with its hosts. The presence of putative virulence-associated genes and antibiotic resistance genes in the viral genomes was assessed using VirulenceFinder ver. 1.5 (https://cge.cbs.dtu.dk/services/VirulenceFinder/) and ResFinder ver. 3.0 (https://cge.cbs.dtu.dk/services/ResFinder/), respectively, with setting described in Kleinheinz et al. (2014).

## Results

### Presence of Prophage-Like Sequences in *Dickeya* spp. and *Pectobacterium* spp. Complete Genomes

**Figure 2:**
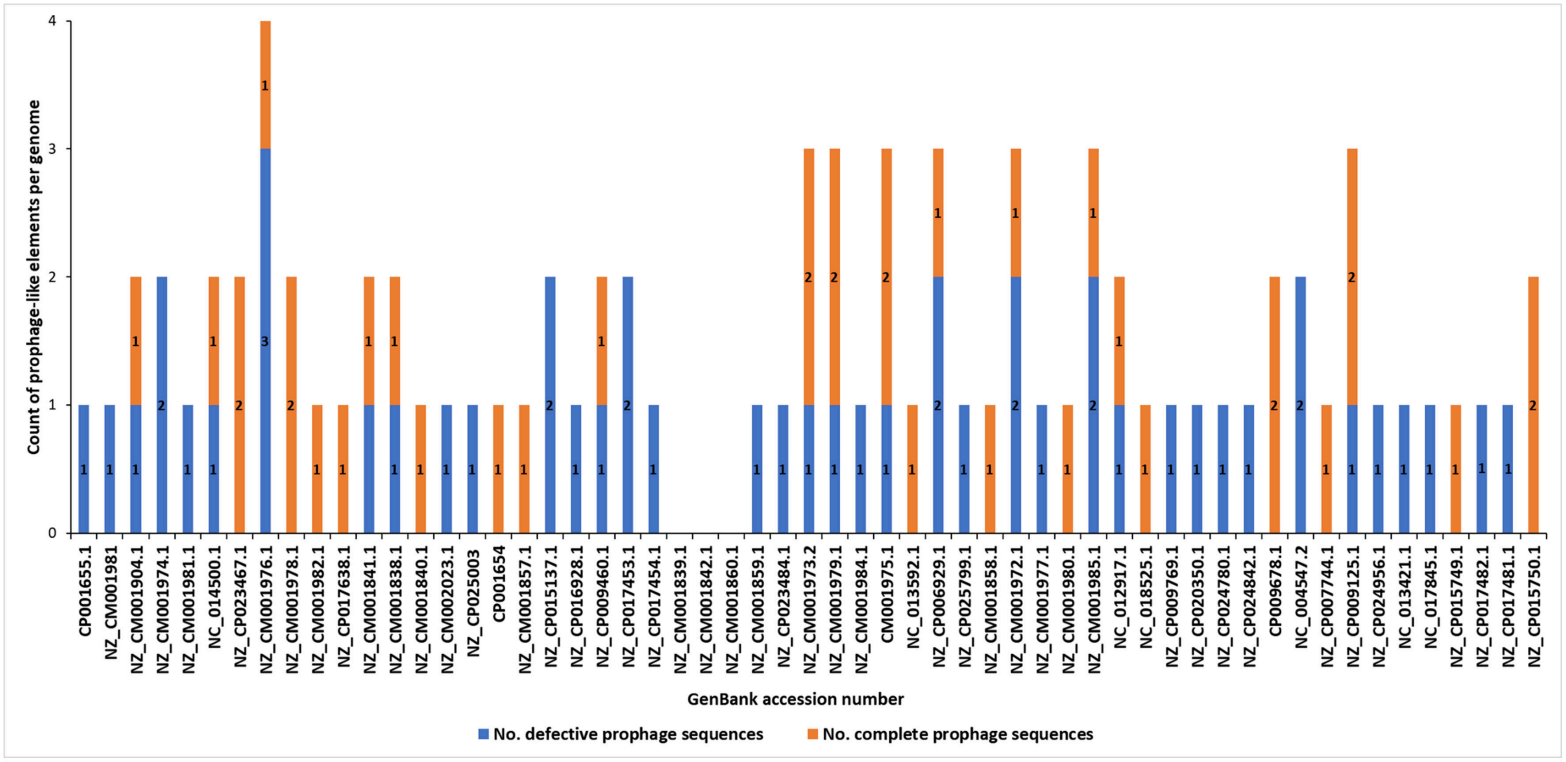
Distribution of putatively defective prophage-like elements and seemingly intact prophages in 57 complete genomes of Soft Rot *Pectobacteriaceae* acquired from GenBank (NCBI). The prophage sequences were detected using PHASTER and PhiSpy and manually curated using BLAST (NCBI) using the pipeline presented in the Figure 1.

The analyses of the 57 complete SRP genomes accessed from GenBank (NCBI) and interrogated with PHASTER and PhiSpy (Figure 1) resulted in discovery of the prophage-like elements in the genomes of 54 of these strains (95% of the genomes interrogated). In total, 37 seemingly intact and 48 putatively defective prophages were found among these strains (Figure 2; Table 1; Supplementary Table 1). Only three *D. solani* genomes, namely *D. solani* strain MK10 (NZ_CM001839.1), *D. solani* strain MK16 (NZ_CM001842.1), and *D. solani* strain GBC 2040 (NZ_CM001860.1) did not harbor any prophage-like elements.

Incomplete (putatively defective) prophage-like elements were present in the majority of the SRP genomes and ranged in size from 4.5 to 41 kb. Often more than one such an element was found in a given strain, as for example, in *D. chrysanthemi* strain NCPPB 402 harboring 2 putatively defective prophages, *D. dadantii* strain NCPPB 898 harboring 3 putatively defective prophages, and *P. atrosepticum* strain SCRI1043 with 2 such prophages.

Seemingly complete (intact) prophage regions were found in 27 *Dickeya* strains while 10 *Pectobacterium* spp. apparently harbored such prophage genomes (Figure 2; Table 1; Data sheets 1, 2). More than one complete prophage region was found in eight SRP (3 *Pectobacterium* spp. and 5 *Dickeya* spp.) genomes (Figure 2).

The sizes of complete prophage genomes varied from 29 to 78 kb and, on average, these viruses comprised between 0.6 to 1.8 % of the host chromosome. The integration of the prophages to the host genomes was in majority random (Table 1).

The prophages were integrated near genes coding for stress resistance proteins, transcriptional regulators, enzymes involved in the fundamental bacterial metabolism, two-component systems, transporters as well as coding for hypothetical proteins. Six prophages however *viz*. phiDch1, phiDdd2, phiDsol1, phiD4, phiDze2, and phiDze6 were integrated near the genes coding for pectate lyases, one of the most important virulence factors of SRP.

All of the complete prophage genomes possessed structural components that were typical of phages in the order *Caudovirales* (tailed bacteriophages), enabling the classification of 30 prophages (81%) to the *Myoviridae* family, 5 prophages (13.5%) to the *Siphoviridae* family, and 2 prophages (5.5%) to the *Podoviridae* family (Table 1).

This study did not reveal the presence of non-integrase based forms of lysogeny, such as that of transposable phages or plasmid-based replication. As the workflow included PHASTER and PhiSpy, it could be expected to detect these sorts of phages if they existed in the dataset. Furthermore, three prophages (*viz*. phiDda2, phiDsol1, and phiPc1) present in the genomes of *D. dadantii* DSM 18020, *D. solani* ND14b, and *P. carotovorum* subsp. *odoriferum* BC S7, respectively, share significant similarity with well-characterized temperate bacteriophage ZF40 infecting *Pectobacterium carotovorum* subsp. *carotovorum* (Table 1) (Korol and Tovkach, 2012).

### Phylogenetic Relationships Between Prophages Found in SRP Genomes Based on Single Gene Analyses

**Figure 3:**
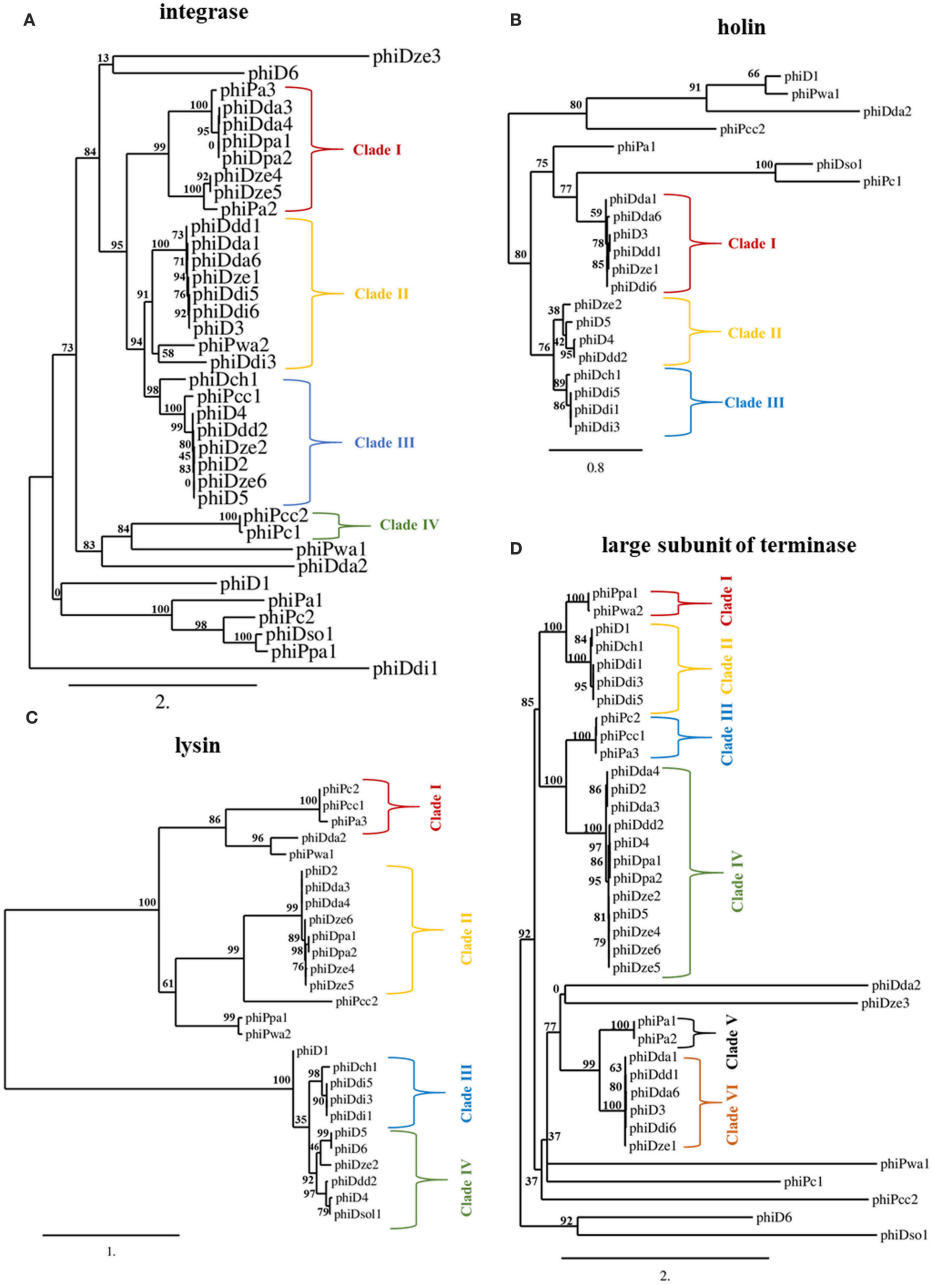
Maximum likelihood (ML) tree based on the aligned amino acid sequences of integrase (present in 37 prophages) **(A)**, holin (present in 21 prophages) **(B)**, lysin (present in 27 prophages) **(C)** and large subunit of terminase (present in 37 prophages) **(D)** genes of seemingly intact prophage sequences distributed in 57 Soft Rot *Pectobacteriaceae* genomes. Phylogenetic studies were performed using Phylogenetic Pipeline of Information Génomique et Structurale, CNRS-AMU, France (http://www.phylogeny.fr/) with bootstrap support for 1,000 replicates. The bar indicates the number of substitutions per sequence position. The cutoff for separating the clades was the bootstrap support for particular branch (n) of at least 70% together with the bootstrap support for particular predecessor branch (n-1) of at least 70%.

The *int* gene encoding integrase and *terL* gene coding for the large subunit of terminase were present in all 37 screened prophages (Figures 3A,D), whereas genes encoding holin (*hol*) and lysin (*lys*) were found within 21 and 27 prophage sequences, respectively (Figures 3B,C). Phylogenetic analyses revealed that SRP prophages are diverse, with viruses belonging to the same viral family forming different phylogenetic clades. The phylogenetic distance between prophages calculated based on the amino acid sequences of integrase did not prove to be useful in determining a phylogenetic association with their host as no clear separation of the *Pectobacterium* and *Dickeya* prophage clades could be observed (Figure 3A).

In contrast, phylogenetic analyses based on amino acid sequences of terminase large subunit, holin, and lysin revealed clades of prophages present in *Dickeya* spp. genomes that were distinct from those in the genomes of *Pectobacterium* spp. strains. This separation of clades was however only partial (Figures 3B–D). Based on terminase amino acid sequences, seven prophage clades could be distinguished each containing between two and twelve viruses. For integrase and lysin amino acid sequences, four prophage clades could be distinguished, each containing between two and nine viruses.

Phylogenetic analysis using holin sequences differentiated three prophage clades. Interestingly, phiDda1, phiDda6, phiD3, phiDdd1, phiDze1, and phiDdi6 were grouped together both in clade I of holin- and in clade II of integrase-based phylogenetic trees while phiDze2, phiD5, phiD4, and phiDdd2 were grouped both in clade III of integrase- and clade II of holin-based tries. Likewise, phiDch1, phiDdi5, phiDdi1, and phiDdi3 were present both in clade III of holin- and clade III of lysin amino acid sequence-based trees.

### Comparative Genomics and Proteomics of SRP Prophages

**Figure 4:**
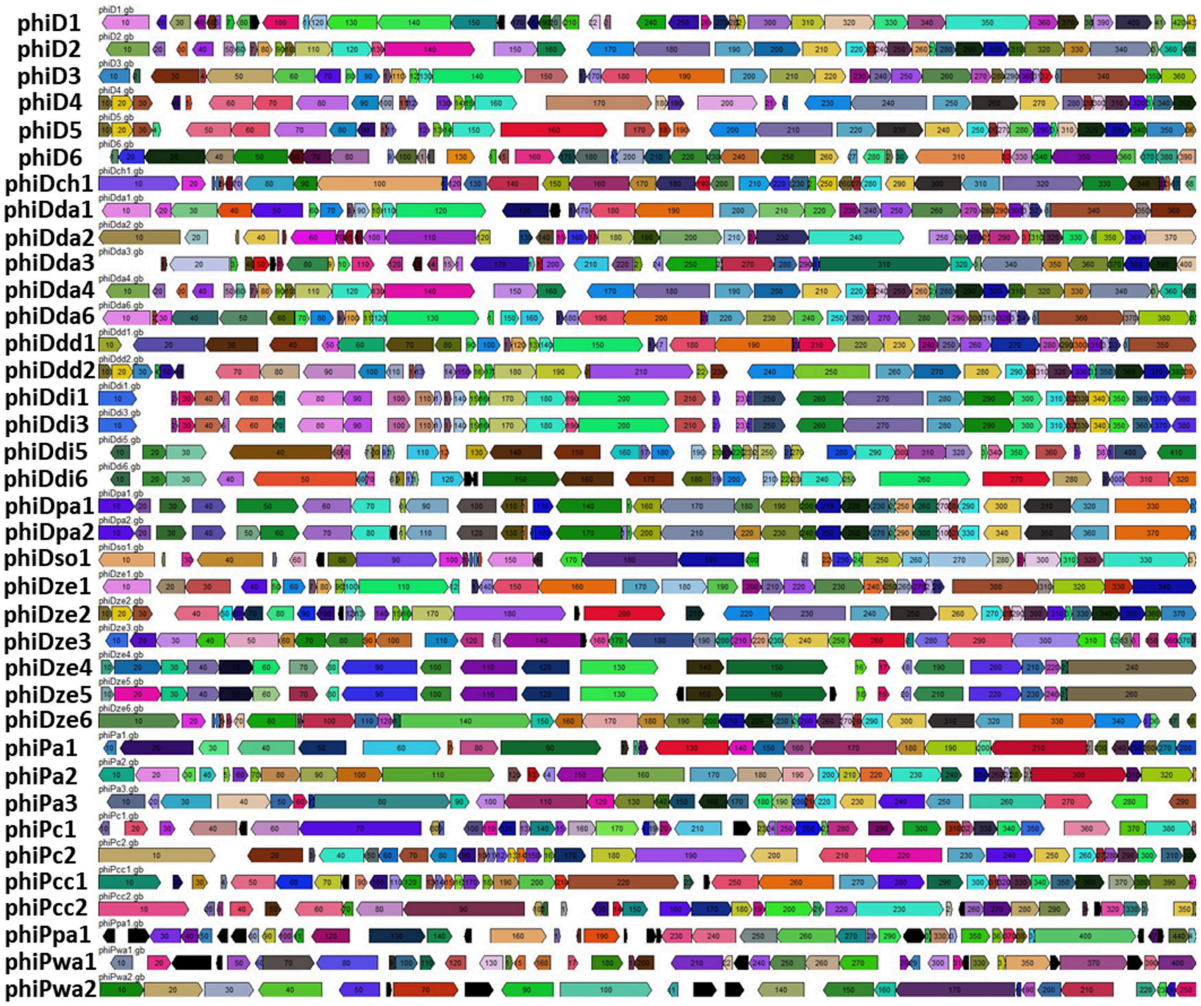
Comparative analyses of the 37 intact prophage genomes. Boxes indicate open reading frames (ORFs) in prophage genomes predicted by RAST annotation pipeline. Homological ORFs are marked with the same color. The analysis and visualization were performed with the use of DNA Master ver. 5.23.2.

Comparative genomics based on the RAST annotated prophage genome sequences allowed visualization of the order of ORFs present in all 37 prophage genomes (Figure 4; Supplementary Figure 1). In general, and with the few exceptions mentioned below, the organization of ORFs within the 37 SRP prophage genomes was not conserved, exhibiting a high genetic mosaicism. The genome organization of only phiDdi1 and phiDdi3 had high synteny while prophage pairs phiDze4 and phiDze5 as well as phiDpa1 and phiDpa2 exhibited somewhat lower conservation of gene order.

The most highly syntenic prophages shared a common host bacterial species; PhiDdi1 and phiDdi3 were found in *D. dianthicola* strains RNS04.9 and NCPPB 453, while phiDze4 and phiDze5 were found in *D. zeae* isolates CSL RW192 and NCPPB 3531, and prophage phiDpa1 and phiDpa2 were found in *D. paradisiaca* strains Ech703 and NCPPB 2511. Only a partial conservation of the order of ORFs was present among phiD4, phiD5, and phiDdd2 (Figure 4) residing in the phylogenetically distinct hosts *Dickeya* sp. NCPPB 3274, *Dickeya* sp. NCPPB 569 and *D. dadantii* subsp. *diffenbachiae* NCPPB 2976. As noted above, bacterial genomes frequently harbored two distinct but complete prophages such as in the case of *D. dadantii* strain DSM 18020 (carrying phiDda2 and phiDda3), *D. dadantii* subsp. *diffenbachiae* strain NCPPB 2976 (carrying phiDdd1 and phiDdd2), *Dickeya* sp. CSL RW240 (carrying phiD1 and phiD2), *Dickeya* sp. Strain NCPPB 3274 (carrying phiD3 and phiD4), *Dickeya* sp. Strain NCPPB 569 (carrying phiD5 and phiD6) and *P. carotovorum* subsp. *odoriferum* strain BC S7 (carrying phiPc1 and phiPc2).

No correlation was observed between the host bacterial genome size and the aggregate prophage genome size (R^2^ = 0.02) (data not shown).

**Figure 5:**
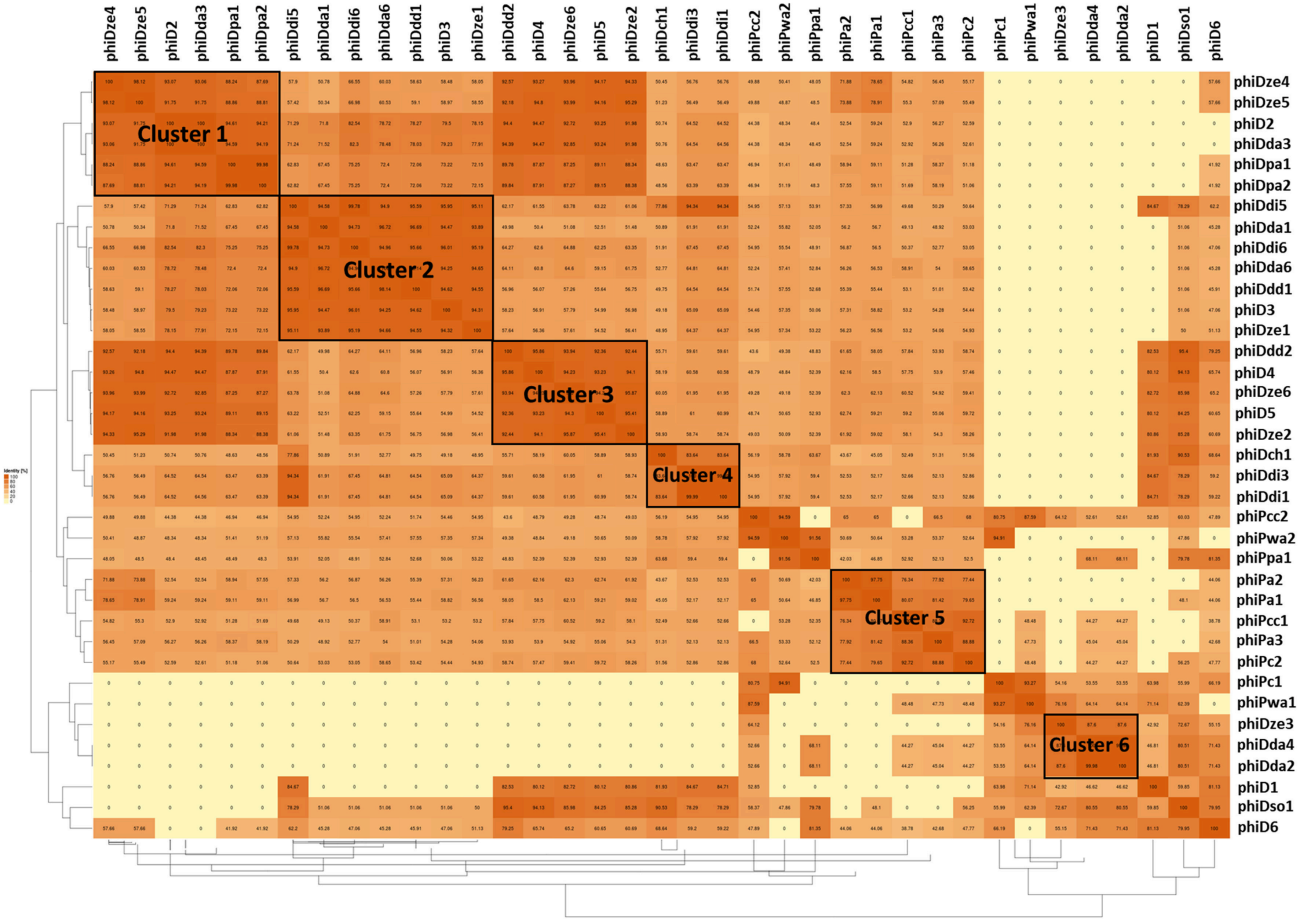
Pairwise average amino acid identity (AAI) heatmap among 37 intact SRP prophages. The map was generated using EDGAR—a software platform for comparative genomics (Blom et al., 2009).

A dot plot matrix constructed based on average amino acid identity (AAI) of the 37 prophage proteomes revealed six visually distinctive clusters (Figure 5); two clusters (Cluster 2 and Cluster 3) (Supplementary Tables 3, 4) having a AAI > 90%, one cluster having a AAI > 85% (Cluster 1) (Supplementary Table 2), two clusters having a AAI > 80% (Cluster 4 and Cluster 6) (Supplementary Tables 5, 7), and one cluster with a AAI only > 75% (Cluster 5) (Supplementary Table 6) Five clusters (Cluster 1, 2, 3, 4, and 6) were grouping proteomes of prophages present in *Dickeya* spp. genomes, whereas Cluster 5 was grouping prophages hosted by *Pectobacterium* spp. strains as evidenced by the AAI dot plot matrix.

### Presence of Unique Genes of Bacterial Origin in the Seemingly Intact Prophage Genomes

**Figure 6:**
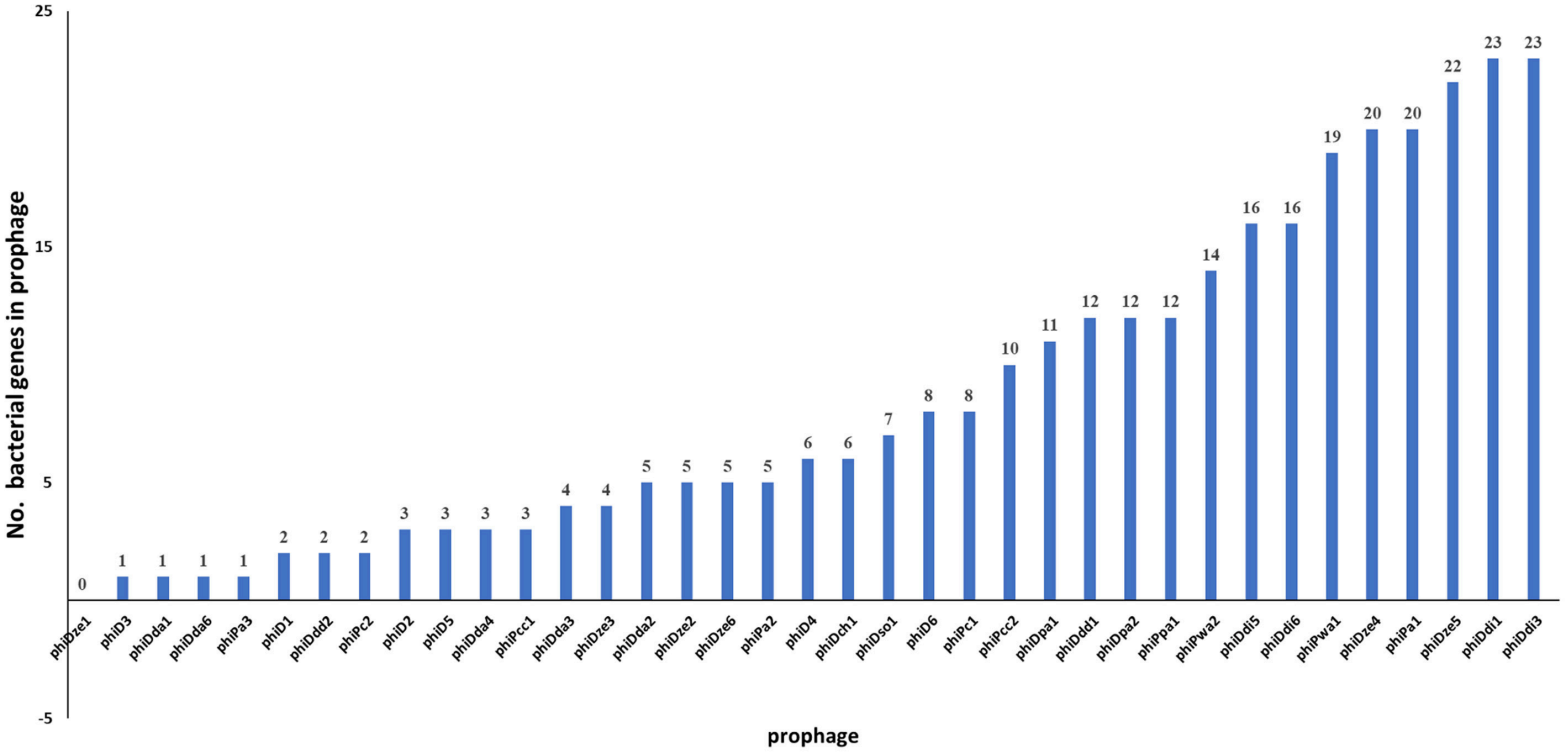
Distribution of genes of bacterial origin in 37 seemingly intact prophages of SRP. The particular gene found in the prophage was classified as being of bacterial origin when altogether: (i) the gene is frequently present in bacterial genome(s), (ii) is unnecessary to complete bacteriophage life cycle, (iii) encodes protein with an enzymatic activity not required by the virus to interact with its hosts.

Of 37 screened complete prophage genomes, only one, phiDze1 did not contain any ORFs of bacterial origin. The other 36 prophages contained between 1 (phiD3, phiDda1) and 23 (phiDdi1 and phiDdi3) ORFs apparently acquired from bacterial hosts (Figure 6). Most of the bacterial ORFs found in prophages encoded proteins involved in primary bacterial metabolism, proteins associated with DNA/RNA repair, energy transfer, DNA/protein regulation and modification and proteins that may be involved in niche exploitation (e.g., resistance to metal ions, nitrogen assimilation, heat shock proteins) (Supplementary Table 8).

The genes present among the large proportion of the seemingly intact prophages were those encoding: (i) methyl-directed repair DNA adenine methylase (in 21 prophage genomes), (ii) methyl-transferase (in 9 prophages), and (iii) modification methylase ScrFIA (in 6 prophages). Interestingly, similar sets of bacterial genes were found in different groups of prophages such as (1) phiD2, phiDda3, and phiDda4, (2) phiDdi1 and phiDdi3, (3) phiDdi5 and phiDdi6, (4) phiDpa1 and phiDpa2, (5) phiDze4 and phiDze5, and (6) phiPc2 and phiPcc1 (Supplementary Table 2). Prophages phiD6, phiDdi1, phiDdi1, and phiPa1 all harbored homologous genes encoding the tellurite resistance protein TerB, while prophages phiDpa1 and phiDpa2 all carried the gene for the cation-efflux pump FieF that confers resistance to cobalt, zinc and cadmium ions.

None of the prophages apparently harbored genes encoding antibiotic resistance genes and genes coding for allergens/toxins when analyzed by VirulenceFinder and ResFinder and by manual inspection with BLAST, and only phiPpa1 contained a gene potentially involved in biosynthesis of a putatively antagonistic factor (monooxygenase antibiotic).

## Discussion

Despite the fact that the majority of bacterial genomes deposited in international genomic sequence databases reveal that phage DNA is commonly integrated into the host chromosome (Canchaya et al., 2003), little is known of how commonly such viruses infect SRP and the extent to which viruses might be associated with virulence or host range of this important group of bacteria (Varani et al., 2013; Czajkowski, 2016).

Initial studies of bacteriophages in this group (*Erwinia chrysanthemi* 3937 phage phiEC2) were reported only in 1984 (Resibois et al., 1984), and while this phage has been widely used since then for generalized transduction of *Dickeya* spp. even it has yet to be characterized in detail and little is known about its ecological, genomic and morphological features (for review see: Czajkowski, 2016). While other temperate SPR bacteriophages have been recently described (for review see: Czajkowski, 2016), little molecular detail is known of these viruses. This study was designed to explain to genome sequence is available for SRP to better understand the frequency of occurrence, diversity, and possible functions of viruses in this group of bacteria.

In this study all available 57 (as of August 2018) *Pectobacterium* spp. and *Dickeya* spp. complete genome sequences present in NCBI GenBank were screened for the presence of prophage-like elements. The *in silico* workflow used here (Figure 1) allowed the identification of prophages in 95% of SRP genomes. Although prophages are known to constitute even as much as 10 to 20% of a bacterial genome, all of the prophages analyzed comprised on average < 2% of the *Pectobacterium* spp. and *Dickeya* spp. chromosome. It is noteworthy that the related foodborne pathogen *Escherichia coli* O157:H7 strain Sakai that can sometimes be found in the same habitats as SRP harbors much more abundant prophages (16% of its total genome content) (Hayashi et al., 2001).

The majority of the SRP prophages (48 sequences) were putatively defective and did not apparently contain those genes essential for bacteriophage interaction with their bacterial hosts such as integrases and genes coding for viral structural proteins. Similarly, some of the screened bacterial genomes were also missing putative attachment sites for these prophage. The frequent occurrence of incomplete prophages in bacterial chromosomes has been reported for various bacteria, including human and animal pathogens as well as for saprophytic bacteria present in soil and water (Casjens, 2003; Bobay et al., 2014). It is widely accepted that bacterial hosts under natural conditions are continuously exposed to phage infections and that some of these events may result in long-term and irreversible phage-bacterial associations on a genomic level (Touchon et al., 2014). This is clearly the case for *Pectobacterium* spp. and *Dickeya* spp. since these strains have a worldwide distribution (Pérombelon, 2002). The high number of putatively defective prophage sequences reported here may further indicate an initial rapid inactivation of viable prophages in bacterial genome is followed by a slow decay of prophage genes due to the accumulations of point mutations and deletions. This so-called phage domestication has been reported for other *Enterobacteriaceae* as a way to cure bacterial genomes from the presence of unnecessary and/or toxic genetic material (Bobay et al., 2014).

The 37 complete prophages found in 29 genomes of *Pectobacterium* spp. and *Dickeya* spp. were characterized in detail. Bioinformatic analysis of the prophage genes encoding viral structural proteins allowed classification of these prophages into different families of the order *Caudovirales* (tailed bacteriophages) with the SRP prophages of the *Myoviridae* family being the most abundant (81% of found prophages). The order *Caudovirales* contains more than 97% of all described phages known to infect bacteria with at least 350 distinct phage isolates documented as members of this order to date (Ackermann, 1998; Fokine and Rossmann, 2014). The great majority of existing bioinformatic tools created to analyze bacteriophage genomes have been developed based on the known *Caudovirales* sequences and consequently they may not be well-suited to analyze viral genomes belonging to different orders and/or groups. Additionally, more than 99% of all SRP bacteriophages described so far also belong to the order *Caudovirales* and occur in three families namely *Myoviridae, Podoviridae*, and *Siphoviridae* (Czajkowski, 2016).

The genome organization and ORF arrangements was not well-conserved across the 37 seemingly intact prophages. The exceptions were the 3 prophage pairs (phiDdi1 and phiDdi3, phiDpa1 and phiiDpa2, and phiDze4 and phiDze5) that were highly conserved with respect to each other. This indicates that overall, SRP prophages are likely mobile, often being transferred between different hosts and easily undergoing rearrangements. It is well-established that prophages are often highly mosaic and that their genomes constitute modules that can be interchanged between different phages by recombination (Hendrix et al., 2000). As it is believed now, such constant recombination events and the resulting mosaicism are the major driving force both for bacteriophage and bacterial evolution (Hendrix et al., 1999; Pedulla et al., 2003).

No linkage was seen between the presence of particular seemingly intact prophages and bacterial genera, bacterial genome size, geographical location, or the environments from which the host bacteria were initially isolated. Likewise, due to the absence of universal genes in bacteriophages that can be used for phylogenetic studies (similar to 16S *rDNA* gene in bacteria), (pro)phage classification is difficult (Lawrence et al., 2002). In this study, contrary to the studies performed earlier in which the usefulness of integrase, holin, and lysin sequences for the phylogenetic studies of prophages were evaluated (Brüssow et al., 2004; Ventura et al., 2005, 2007), none of these genes proved useful for clear phylogenetic separation of prophages of *Pectobacterium* spp. and *Dickeya* spp. into distinct clades. Such a lack of phylogenetic association suggests an independent evolution of prophages and their SRP hosts (Colavecchio et al., 2017). This is perhaps not a surprise given that SRP are not only naturally present in many and widely different environments (e.g., soil, water, plant surface, on and inside insects) but are often dispersed from one environment to another (Perombelon, 1988; Charkowski, 2006). All these lifestyle changes would require a rapid adaptation to a new setting, a process that might not facilitate stable association of phage with a given habitat or host (Ma et al., 2007; Reverchon et al., 2016).

More than 50% of complete prophage genomes contained not only the genes encoding structural viral proteins and integrases, but also genes coding for holin and lysin. Additionally, the 13 prophages (35% of the complete prophages) contained genes encoding both proteins. Lysins and holins are viral enzymes leading to disruption of the host cells and enabling propagation of bacteriophages in the environment (Wang et al., 2000). As both holin and lysin are viewed as facilitating host infection (Young, 2014), the presence of these genes in SRP prophages may give the first assumption that those viruses may be more infective than the prophages lacking one or both genes (Feiner et al., 2015). This further indicates that in at least these 13 prophages may be possibly easily induced, thus it they may become transmittable upon encounter of particular environmental stimuli (Nanda et al., 2014). However, the point must be made that without the further experiments, the infectivity of the mentioned prophages remains rather speculative at the moment.

Likewise, the absence of holin and/or lysin or both genes in the phage genome does not necessarily characterize a bacteriophage as harmless. For example, the well-characterized infectious *Dickeya* spp. bacteriophage LIMEstone1 (Adriaenssens et al., 2012) and ϕD5 (Czajkowski et al., 2014) both lack the gene coding for holin, and the *P. carotovorum* subsp. *carotovorum* phage PP1 lacks the gene coding for lysin (Lee et al., 2012).

It seems likely that induction of SRP prophages will have an impact on environmental fitness and virulence of the hosts. An understanding of the conditions in which lysis is induced might make it possible to achieve some level of control of the diseases caused by these SRP by appropriately modifying the environment. Alternatively, it can be speculated that the newly found holin and lysin genes, produced at an industrial scale might be a useful tool in the biological control of such diseases as previously suggested (Fenton et al., 2010).

The high abundance of (seemingly intact) prophages in the SRP genomes may have as well a direct impact on control of *Pectobacterium* spp. and *Dickeya* spp. in agricultural applications. Prophages are known to utilize mechanism called superinfection exclusion which prevents subsequent viral infections of the same hosts (Bondy-Denomy and Davidson, 2014). It can be speculated that effectiveness of biological control of SRP with the use of lytic bacteriophages may be reduced due to the prophage-induced resistance in the target bacteria. The superinfection exclusion has been analyzed in detail in several human pathogenic bacteria including *Escherichia coli, Pseudomonas aeruginosa* and *Salmonella* spp. (for review see: Labrie et al., 2010), its ecological role has never been however assessed in SRP. Considering the increasing interest in phage therapy as a means to combat plant pathogenic bacteria, and specifically SRP, this topic undoubtedly needs further examination.

Based on average amino acid identity (AAI), six prophage clusters; five present in *Dickeya* spp. and one within *Pectobacterium* spp. could be identified in this large collection of strains. As opposed to the phylogenetic analyses based on a single given prophage gene, AAI appears to be a more powerful method to phylogenetically separate the prophages residing in the genomes of *Pectobacterium* spp. and *Dickeya* spp. While AAI has been suggested to be a better phylogenetic method for whole genome-based taxonomy of *Prokaryotes* (Konstantinidis and Tiedje, 2005), this method has received little usage in the phylogenetic analysis of viruses. The presented results suggest that it would prove useful in bioinformatics analyses of prophage such as in this study.

It is well-established that prophages often encode genes that are not directly involved in viral propagation and infection but which can confer a fitness benefit to their hosts (Bondy-Denomy and Davidson, 2014). These genes can enhance the virulence of the bacteria directly by prophage-encoded toxins and/or indirectly by increasing bacterial fitness which indirectly results in enhanced virulence (Hacker and Carniel, 2001). All but one of the 37 seemingly intact prophages described in this study contained at least one gene that was apparently acquired from other host bacteria (probably from *Dickeya* spp. and *Pectobacterium* spp. strains or their close relatives), as a result of infection of one or more previous hosts. Likewise, the majority of prophages analyzed in this study contained multiple genes of bacterial origin, with two prophages phiDdi1 and phiDdi3 carrying even as many as 23 bacterial genes. It remains unclear however whether the bacterial genes found in these prophage genomes are transcribed or translated. Surprisingly, several prophages present in different bacterial genomes carried homological set of bacterial genes indicating that possibly these prophages propagated in co-occurring host populations of different species at the same time. None of the 36 prophages analyzed here however acquired bacterial genes encoding well-described virulence factors exploited by *Pectobacterium* spp. and *Dickeya* spp. to infect plants (Reverchon and Nasser, 2013). Instead, the prophages carried genes that may apparently contribute to ecological fitness in complex and diverse environments; e.g., genes encoding metal ion transporters, enzymes involved in energy metabolism, heat shock proteins, nitrogen assimilation proteins as well as genes coding for DNA methylases which may be used in protecting prophage sequences in the host genome from excision by changing DNA methylation pattern (Canchaya et al., 2003). This may as well-explain the high number of prophage sequences observed in many bacterial genomes (Ohnishi et al., 2001; Matos et al., 2013) and the relatively high proportion of prophage-related genes in pathogenic strains in comparison with saprophytic, non-pathogenic bacteria (Busby et al., 2013). The most common gene present in seemingly intact prophage genomes was one encoding methyl-directed repair DNA adenine methylase (EC 2.1.1.72), being found in 21 viruses. This is a large group of enzymes that apart from being members of restriction-modification systems of many Gram-negative bacteria, plays important roles in regulation of genes encoding virulence factors in bacterial pathogens at the posttranscriptional level (Marinus and Casadesus, 2009). Unfortunately their role, if any, in pathogenicity of SRPs remains cryptic.

The biggest limitation of the *in silico* workflow used here is obviously that the classification of prophage element to the group of intact or defective prophages and their impact on the host fitness is based on the genome data alone. However, in general, the relatively high number of seemingly intact prophages found in the study suggest that the interaction of SRP and bacteriophages in the natural environment may be highly significant for the ecology, adaptation, and evolution of *Pectobacterium* spp. and *Dickeya* spp. Prophage induction experiments are now being conducted to further elucidate the role of prophages present in SRP strains and to better understand the molecular basis of (pro)phage-bacteria interactions.

## Author Contributions

RC: conceptualization, data curation, formal analysis, funding acquisition, investigation, methodology, project administration, resources, supervision, validation, visualization, writing and original draft, writing and review, and editing.

### Conflict of Interest Statement

The author declares that the research was conducted in the absence of any commercial or financial relationships that could be construed as a potential conflict of interest.
