# Supplementary material for: May the Phage be With You? Prophage-Like Elements in the Genomes of Soft Rot Pectobacteriaceae: Pectobacterium spp. and Dickeya spp
Source: Front Microbiol. 2019 Feb 14;10:138. doi: 10.3389/fmicb.2019.00138 (PMC6385640; doi:10.3389/fmicb.2019.00138)
Supplement: Supplementary file 3 [file Data_Sheet_3.PDF]

Supplementary Material

May the phage be with you? Prophage-like elements in the genomes of Soft Rot *Pectobacteriaceae*: *Pectobacterium* spp. and *Dickeya* spp.

Robert Czajkowski *

University of Gdansk, Intercollegiate Faculty of Biotechnology, University of Gdansk and Medical University of Gdansk, Laboratory of Biologically Active Compounds, A. Abrahama 58, 80-307 Gdansk, Poland

∗ Correspondence:

Robert Czajkowski

Robert.Czajkowski@biotech.ug.edu.pl

Supplementary Figure 1. Comparative analyses of the 37 intact prophage genomes.
Multiple genome alignment of the 37 seemingly intact prophages was performed using Mauve software (http://asap.ahabs.wisc.edu/mauve/). phiD1 gene coding for integrase is the reference for alignments and comparisons to the other 36 prophages. Boxes with identical colors represent local colinear blocks (LCB), indicating homologous DNA regions shared by two or more prophages without sequence rearrangements.

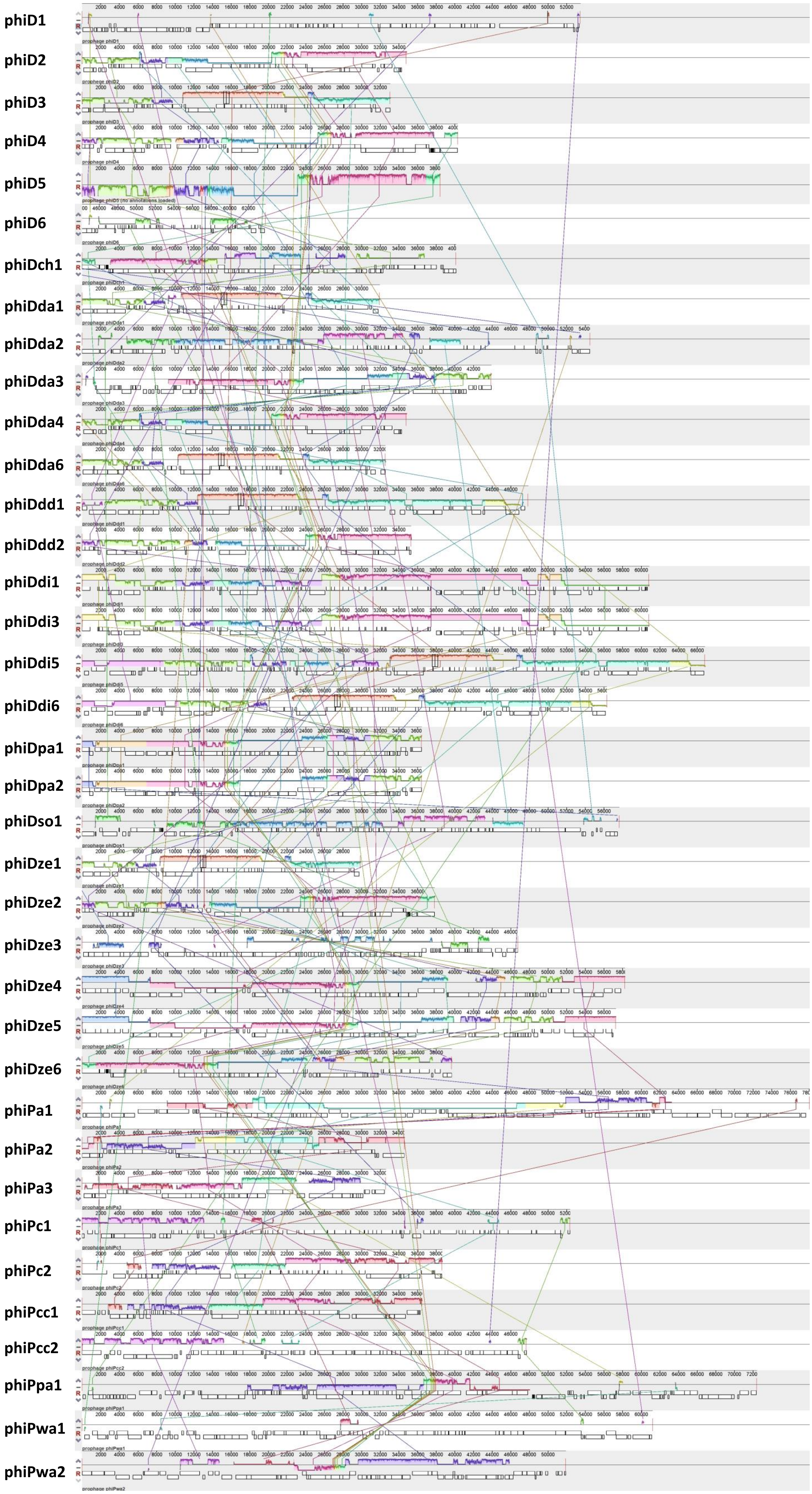
phiD1
phiD2
phiD3
phiD4
phiD5
phiD6
phiDch1
phiDda1
phiDda2
phiDda3
phiDda4
phiDda6
phiDdd1
phiDdd2
phiDdi1
phiDdi3
phiDdi5
phiDdi6
phiDpa1
phiDpa2
phiDso1
phiDze1
phiDze2
phiDze3
phiDze4
phiDze5
phiDze6
phiPa1
phiPa2
phiPa3
phiPc1
phiPc2
phiPcc1
phiPcc2
phiPpa1
phiPwa1
phiPwa2
