# Supplementary material for: May the Phage be With You? Prophage-Like Elements in the Genomes of Soft Rot Pectobacteriaceae: Pectobacterium spp. and Dickeya spp
Source: Front Microbiol. 2019 Feb 14;10:138. doi: 10.3389/fmicb.2019.00138 (PMC6385640; doi:10.3389/fmicb.2019.00138)
Supplement: Supplementary file 4 [file Data_Sheet_4.PDF]

Supplementary Material

May the phage be with you? Prophage-like elements in the genomes of Soft Rot *Pectobacteriaceae*: *Pectobacterium* spp. and *Dickeya* spp.

Robert Czajkowski *

University of Gdansk, Intercollegiate Faculty of Biotechnology, University of Gdansk and Medical University of Gdansk, Laboratory of Biologically Active Compounds, A. Abrahama 58, 80-307 Gdansk, Poland

∗ Correspondence:

Robert Czajkowski

Robert.Czajkowski@biotech.ug.edu.pl

Supplementary Table 1. Genomic features of putatively defective prophages (prophage-like elements) present in the complete genomes of Soft Rot Pectobacteriaceae (Dickeya spp. and Pectobacterium spp.) obtained from GenBank (NCBI)

Supplementary Table 1. Genomic features of putatively defective prophages (prophage-like elements) present in the complete genomes of Soft Rot *Pectobacteriaceae* (*Dickeya* spp. and *Pectobacterium* spp.) obtained from GenBank (NCBI)

No	Putatively defective prophage	Host (GenBank accession)	Prophage size (kb)	Coordinates in bacterial genome	Reason(s) to classify the prophage as putatively defective virus
**1**	dev_phiDch1	*Dickeya chrysanthemi* Ech1591 (CP001655.1)	27.2	1,726,935-1,754,156	PHASTER score: defective prophage<br>PhiSpy score: defective prophage<br><br>Manual inspection:<br>structural phage proteins - absent, terminase - absent, hypothetical proteins inside prophage (no phage proteins from RAST annotation)
**2**	dev_phiDch2	*Dickeya chrysanthemi* NCPPB 3533 (NZ_CM001981)	11.4	4,394,289-4,405,771	PHASTER score: defective prophage<br>PhiSpy score: defective prophage<br><br>Manual inspection:<br>structural phage proteins - absent, terminase - absent hypothetical proteins inside prophage (no phage proteins from RAST annotation)
**3**	dev_phiDch3	*Dickeya chrysanthemi* NCPPB 516 (NZ_CM001904.1)	28.9	1,434,666-1,463,629	PHASTER score: defective prophage<br>PhiSpy score: defective prophage<br><br>Manual inspection:<br>structural phage proteins - absent,<br>terminase - absent,<br>hypothetical proteins inside prophage (no phage proteins from RAST annotation)
**4**	dev_phiDch4	*Dickeya chrysanthemi* NCPPB 402 (NZ_CM001974.1)	14.7	1,903,705-1,918,432	PHASTER score: defective prophage<br>PhiSpy score: defective prophage<br><br>Manual inspection:

					structural phage proteins - absent,<br>terminase - absent,<br>hypothetical proteins inside prophage (no phage proteins from RAST annotation) and prophage-like proteins
**5**	dev_phiDch5	*Dickeya chrysanthemi* NCPPB 402<br>(NZ_CM001974.1)	16	3,044,617-<br>3,060,670	PHASTER score: defective prophage<br>PhiSpy score: defective prophage<br><br>Manual inspection:<br>structural phage proteins - absent,<br>terminase - absent,<br>hypothetical proteins inside prophage (no phage proteins from RAST annotation) and prophage-like proteins
**6**	dev_phiDch6	*Dickeya chrysanthemi* NCPPB 3533<br>(NZ_CM001981.1)	27.2	1,674,108-<br>1,701,317	PHASTER score: defective prophage<br>PhiSpy score: defective prophage<br><br>Manual inspection:<br>structural phage proteins - absent,<br>terminase - absent,<br>hypothetical proteins inside prophage (no phage proteins from RAST annotation) and prophage-like proteins
**7**	dev_phiDda1	*Dickeya dadantii* 3937<br>(NC_014500.1)	8.8	1,988,302-<br>1,997,171	PHASTER score: defective prophage<br>PhiSpy score: defective prophage<br><br>Manual inspection:<br>structural phage proteins - absent,<br>terminase - absent,<br>integrase - absent,<br>attachment sites - absent,<br>hypothetical proteins inside prophage (no phage proteins from RAST annotation) and prophage-like proteins, very small genome
**8**	dev_phiDda2	*Dickeya dadantii* NCPPB 898<br>(NZ_CM001976.1)	6	1,637,361-<br>1,643,411	PHASTER score: defective prophage<br>PhiSpy score: defective prophage

					Manual inspection:<br>structural phage proteins - absent,<br>terminase - absent,<br>integrase - absent,<br>attachment sites - absent,<br>hypothetical proteins inside prophage (no phage proteins from RAST annotation), very small genome
**9**	dev_phiDda3	*Dickeya dadantii* NCPPB 898 (NZ_CM001976.1)	17	3,062,208-3,079,278	PHASTER score: defective prophage<br>PhiSpy score: intact prophage<br><br>Manual inspection:<br>structural phage proteins - absent,<br>terminase - absent,<br>hypothetical proteins inside prophage (no phage proteins from RAST annotation) and prophage-like proteins
**10**	dev_phiDda4	*Dickeya dadantii* NCPPB 898 NZ_CM001976.1	11.9	212,385-224,310	PHASTER score: intact<br>PhiSpy score: intact<br><br>Manual inspection:<br>terminase - absent,<br>integrase - absent,<br>attachment sites - absent,<br>no plate proteins, no tRNAs
**11**	dev_phiDdi1	*Dickeya dianthicola* NCPPB 453 (NZ_CM001841.1)	14	2,881,443-2,895,497	PHASTER score: defective prophage<br>PhiSpy score: intact prophage<br><br>Manual inspection:<br>terminase - absent,<br>integrase - absent,<br>structural phage proteins - absent,<br>only phage-like proteins present,<br>one attachment site inside prophage
**12**	dev_phiDdi2	*Dickeya dianthicola* GBBC 2039	21.1	2,762,233-2,783,403	PHASTER score: defective prophage<br>PhiSpy score: defective prophage

		(NZ_CM001838.1)			Manual inspection:<br>terminase - absent,<br>structural phage proteins - absent,<br>only phage-like proteins present
**13**	dev_phiDdi3	*Dickeya dianthicola* IPO 980<br>(NZ_CM002023.1)	14	2,859,873-<br>2,873,927	PHASTER score: defective prophage<br>PhiSpy score: intact prophage<br><br>Manual inspection:<br>terminase - absent,<br>integrase - absent,<br>structural phage proteins - absent,<br>only phage-like proteins present,<br>one attachment site inside prophage
**14**	dev_Dfa1	*Dickeya fangzhongdai* DSM 101947<br>(NZ_CP025003)	41	1,078,600-<br>1,119,653	PHASTER score: defective prophage<br>PhiSpy score: defective prophage<br><br>Manual inspection:<br>terminase - absent,<br>integrase - absent,<br>structural phage proteins - absent,<br>only phage-like proteins present,<br>several attachment sites inside prophage
**15**	dev_phiDso1	*Dickeya solani* IPO 2222<br>(NZ_CP015137.1)	7.4	1,903,206-<br>1,910,669	PHASTER score: defective prophage<br>PhiSpy score: defective prophage<br><br>Manual inspection:<br>terminase - absent,<br>integrase - absent,<br>structural phage proteins - absent except several fiber proteins,<br>attachment sites absent,<br>very small genome
**16**	dev_phiDso2	*Dickeya solani* IPO 2222<br>(NZ_CP015137.1)	17.4	4,064,340-<br>4,081,806	PHASTER score: defective prophage<br>PhiSpy score: defective prophage

					Manual inspection:<br>terminase - absent,<br>structural phage proteins - absent,<br>attachment sites - absent,<br>hypothetical proteins inside prophage (no phage proteins from RAST annotation) and prophage-like proteins,<br>very small genome
**17**	dev_phiDso3	*Dickeya solani* RNS 08.23.3.1.A (NZ_CP016928.1)	7.4	2,563,956-2,571,419	PHASTER score: defective prophage<br>PhiSpy score: defective prophage<br><br>Manual inspection:<br>terminase - absent,<br>integrase - absent,<br>structural phage proteins absent except several fiber proteins,<br>attachment sites - absent,<br>several hypothetical proteins (no phage proteins from RAST annotation),<br>very small genome
**18**	dev_phiDso4	*Dickeya solani* ND14b (NZ_CP009460.1)	25.5	1,872,724-1,898,303	PHASTER score: defective prophage<br>PhiSpy score: defective prophage<br><br>Manual inspection:<br>terminase - absent,<br>structural phage proteins - absent,<br>attachment site inside prophage,<br>several hypothetical proteins (no phage proteins from RAST annotation)
**19**	dev_phiDso5	*Dickeya solani* D s0432-1 (NZ_CP017453.1)	7.4	3,006,991-3,014,454	PHASTER score: defective prophage<br>PhiSpy score: defective prophage<br><br>Manual inspection:<br>terminase - absent,

					structural phage proteins absent except fiber proteins,<br>attachment sites - absent,<br>very small genome
**20**	dev_phiDso6	*Dickeya solani* D s0432-1 (NZ_CP017453.1)	11.8	4,901,564-4,913,366	PHASTER score: intact prophage<br>PhiSpy score: defective prophage<br><br>Manual inspection:<br>terminase - absent,<br>integrase - absent,<br>structural phage proteins absent except fiber proteins and tail proteins,<br>attachment sites - absent
**21**	dev_phiDso7	*Dickeya solani* PPO 9019 (NZ_CP017454.1)	7.4	3,000,207-3,007,670	PHASTER score: defective prophage<br>PhiSpy score: intact prophage<br><br>Manual inspection:<br>terminase - absent,<br>structural phage proteins absent except fiber proteins,<br>attachment sites - absent,<br>very small genome
**22**	dev_phiDso8	*Dickeya solani* IPO 2222 (NZ_CM001859.1)	14.1	159,329-173,448	PHASTER score: defective prophage<br>PhiSpy score: defective prophage<br><br>Manual inspection:<br>terminase - absent,<br>integrase - absent,<br>structural phage proteins absent except one fiber protein,<br>attachment sites - absent,<br>several hypothetical proteins (no phage proteins from RAST annotation) and phage-like proteins
**23**	dev_phiD1	*Dickeya* sp. Secpp 1600 (NZ_CP023484.1)	11.3	3,482,432-3,493,788	PHASTER score: defective prophage<br>PhiSpy score: defective prophage

					Manual inspection:<br>terminase - absent,<br>structural phage proteins absent except two coat proteins,<br>several phage-like proteins (no phage proteins from RAST annotation)
**24**	dev_phiD2	*Dickeya* sp. CSL RW240 (NZ_CM001973.2)	9.2	4,051,182-4,060,383	PHASTER score: intact prophage<br>PhiSpy score: defective prophage<br><br>Manual inspection:<br>terminase - absent,<br>integrase - absent,<br>structural phage proteins absent except several fiber proteins,<br>attachment sites - absent,<br>very small genome
**25**	dev_phiD3	*Dickeya* sp. NCPPB 3274 (NZ_CM001979.1)	4.5	1,267,272-1,271,855	PHASTER score: defective prophage<br>PhiSpy score: intact prophage<br><br>Manual inspection:<br>terminase - absent,<br>integrase - absent,<br>structural phage proteins - absent,<br>attachment sites - absent,<br>very small genome
**26**	dev_phiD4	*Dickeya* sp. MK7 (NZ_CM001984.1)	20.1	977,244-997,372	PHASTER score: defective prophage<br>PhiSpy score: defective prophage<br><br>Manual inspection:<br>terminase - absent,<br>structural phage proteins - absent,<br>one attachment site inside the prophage genome
**27**	dev_phiD5	*Dickeya* sp. NCPPB 569 (CM001975.1)	18.7	406,188-424,979	PHASTER score: defective prophage<br>PhiSpy score: defective prophage

					Manual inspection:<br>terminase - absent,<br>structural phage proteins - absent,<br>several attachment sites inside the prophage genome,<br>several hypothetical proteins (no phage proteins from RAST annotation) and phage-like proteins
**28**	dev_phiDze1	*Dickeya zeae* EC1 (NZ_CP006929.1)	5.3	115,442-120,824	PHASTER score: defective prophage<br>PhiSpy score: defective prophage,<br><br>Manual inspection:<br>terminase - absent,<br>structural phage proteins - absent,<br>attachment sites - absent,<br>several hypothetical proteins (no phage proteins from RAST annotation),<br>very small genome
**29**	dev_phiDze2	*Dickeya zeae* EC1 (NZ_CP006929.1)	26.3	2,446,171-2,472,491	PHASTER score: intact<br>PhiSpy score: defective prophage,<br><br>Manual inspection:<br>terminase - absent,<br>integrase - absent,<br>attachment sites - absent,<br>structural proteins absent except several tail proteins and several fiber proteins
**30**	dev_phiDze3	*Dickeya zeae* MS2 (NZ_CP025799.1)	25.8	2,665,411-2,691,264	PHASTER score: intact<br>PhiSpy score: defective prophage,<br><br>Manual inspection:<br>terminase - absent,<br>integrase - absent,<br>attachment sites - absent,<br>structural proteins absent except several plate proteins and several tail proteins

**31**	dev_phiDze4	*Dickeya zeae* CSL RW192 (NZ_CM001972.1)	35.4	437,531-473,005	PHASTER score: intact<br>PhiSpy score: defective prophage,<br><br>Manual inspection:<br>attachment sites - absent,<br>integrase - absent,<br>very many hypothetical proteins (no phage proteins from RAST annotation)
**32**	dev_phiDze5	*Dickeya zeae* CSL RW192 (NZ_CM001972.1)	38.7	2,147,072-2,185,811	PHASTER score: intact<br>PhiSpy score: defective prophage,<br><br>Manual inspection:<br>terminase - absent,<br>integrase - absent,<br>structural proteins absent except fiber proteins,<br>very many hypothetical proteins, (no phage proteins from RAST annotation)
**33**	dev_phiDze6	*Dickeya zeae* NCPPB 2538 (NZ_CM001977.1)	7.2	721,952-729,209	PHASTER score: defective prophage<br>PhiSpy score: intact,<br><br>Manual inspection:<br>integrase - absent,<br>terminase - absent,<br>attachment sites - absent,<br>several hypothetical proteins (no phage proteins from RAST annotation) and phage-like proteins, structural phage proteins absent,<br>very small genome
**34**	dev_phiDze7	*Dickeya zeae* MK19 (NZ_CM001985.1)	29	1,608,162-1,637,186	PHASTER score: defective prophage<br>PhiSpy score: defective prophage,<br><br>Manual inspection:<br>terminase - absent,<br>structural phage proteins - absent,

					several hypothetical proteins (no phage proteins from RAST annotation) and phage-like proteins
**35**	dev_phiDze8	*Dickeya zeae* MK19 (NZ_CM001985.1)	31.5	2,747,732-2,779,303	PHASTER score: intact prophage<br>PhiSpy score: defective prophage,<br><br>Manual inspection:<br>integrase - absent,<br>terminase - absent,<br>structural phage proteins absent except tail proteins and plate proteins,<br>attachment sites - absent,<br>very many hypothetical proteins (no phage proteins from RAST annotation) and phage-like proteins
**36**	dev_phiPcc1	*Pectobacterium carotovorum* subsp. *carotovorum* PC1 (NC_012917.1)	12.2	833,155-845,434	PHASTER score: defective prophage<br>PhiSpy score: intact,<br>Manual inspection: terminase absent, structural phage proteins absent except coat protein, several hypothetical proteins (no phage proteins from RAST annotation) and phage-like proteins
**37**	dev_phiPcb1	*Pectobacterium carotovorum* subsp. *brasiliense* BC1 (NZ_CP009769.1)	22.9	1,850,244-1,873,234	PHASTER score: defective prophage<br>PhiSpy score: defective prophage,<br><br>Manual inspection:<br>integrase - absent,<br>terminase - absent,<br>structural phage proteins absent except fiber proteins and tail proteins, several phage-like proteins,<br>attachment sites - absent
**38**	dev_phiPcb2	*Pectobacterium carotovorum* subsp. *brasiliense* SX309 (NZ_CP020350.1)	19.2	3,018,770-3,037,979	PHASTER score: defective prophage<br>PhiSpy score: defective prophage,<br><br>Manual inspection:<br>integrase - absent,<br>terminase - absent,

					structural phage proteins absent except fiber proteins and tail proteins, several phage-like proteins,<br>attachment sites - absent
**39**	dev_phiPcb3	*Pectobacterium carotovorum* subsp. *brasiliense* BZA12 (NZ_CP024780.1)	27.1	1,488,901-1,516,076	PHASTER score: defective prophage<br>PhiSpy score: defective prophage,<br><br>Manual inspection:<br>terminase - absent,<br>structural phage proteins - absent,<br>several phage-like proteins, several proteins of unknown origin and function,<br>attachment sites present inside prophage genome
**40**	dev_phiPc1	*Pectobacterium carotovorum* 3-2 (NZ_CP024842.1)	11.2	887,864-899,143	PHASTER score: defective prophage<br>PhiSpy score: defective prophage,<br><br>Manual inspection:<br>terminase - absent,<br>structural phage proteins absent except coat protein,<br>several phage-like proteins
**41**	dev_phiPa1	*Pectobacterium atrosepticum* SCRI1043 (NC_004547.2)	36.1	4,144,591-4,180,770	PHASTER score: intact prophage<br>PhiSpy score: defective prophage,<br><br>Manual inspection:<br>terminase - absent,<br>integrase - absent,<br>structural phage proteins absent except fiber proteins,<br>very many phage-like proteins,<br>attachment sites - absent
**42**	dev_phiPa2	*Pectobacterium atrosepticum* SCRI1043 (NC_004547.2)	40.6	2,926,054-2,966,671	PHASTER score: intact prophage<br>PhiSpy score: defective prophage,<br><br>Manual inspection:<br>integrase - absent,<br>terminase - absent,

					very many phage-like proteins,<br>attachment site present inside prophage
**43**	dev_phiPa3	*Pectobacterium atrosepticum* 21A (NZ_CP009125.1)	52.9	1,954,333-2,007,299	PHASTER score: defective prophage<br>PhiSpy score: defective prophage,<br><br>Manual inspection:<br>terminase - absent,<br>very many phage-like proteins and proteins with unknown function,<br>attachment site present inside prophage genome
**44**	dev_phiPa4	*Pectobacterium atrosepticum* 36A (NZ_CP024956.1)	11.8	3,353,256-3,365,065	PHASTER score: defective prophage<br>PhiSpy score: defective prophage,<br><br>Manual inspection:<br>terminase - absent,<br>very many phage-like proteins and hypothetical proteins,<br>structural proteins absent except the coat proteins
**45**	dev_phiPpa1	*Pectobacterium parmentieri* WPP163 (NC_013421.1)	19.2	3,144,791-3,164,073	PHASTER score: defective prophage<br>PhiSpy score: defective prophage,<br><br>Manual inspection:<br>terminase - absent,<br>integrase - absent,<br>structural phage proteins - absent,<br>attachment sites - absent
**46**	dev_phiPpa2	*Pectobacterium parmentieri* SCC3193 (NC_017845.1)	5.6	3,792,760-3,798,438	PHASTER score: defective prophage<br>PhiSpy score: defective prophage,<br><br>Manual inspection:<br>terminase - absent,<br>structural phage proteins absent except fiber proteins and tail proteins,<br>attachment sites - absent,<br>proteins of unknown functions,

					very small genome
**47**	dev_phiPpo1	*Pectobacterium polaris* NIBIO1392 (NZ_CP017482.1)	19.2	685,174-704,457	PHASTER score: defective prophage<br>PhiSpy score: defective prophage,<br><br>Manual inspection:<br>terminase absent,<br>structural phage proteins absent except fiber proteins,<br>attachment sites absent,<br>proteins of unknown functions,<br>very many phage-like proteins
**48**	dev_phiPpo2	*Pectobacterium polaris* NIBIO1006 (NZ_CP017481.1)	11.2	3,319,162-3,330,385	PHASTER score: defective prophage<br>PhiSpy score: defective prophage,<br><br>Manual inspection:<br>terminase - absent,<br>integrase - absent,<br>structural phage proteins - absent,<br>attachment sites - absent,<br>proteins of unknown functions,<br>very many phage-like proteins
