# Supplementary material for: May the Phage be With You? Prophage-Like Elements in the Genomes of Soft Rot Pectobacteriaceae: Pectobacterium spp. and Dickeya spp
Source: Front Microbiol. 2019 Feb 14;10:138. doi: 10.3389/fmicb.2019.00138 (PMC6385640; doi:10.3389/fmicb.2019.00138)
Supplement: Supplementary file 5 [file Data_Sheet_5.PDF]

Supplementary Material

May the phage be with you? Prophage-like elements in the genomes of Soft Rot *Pectobacteriaceae*: *Pectobacterium* spp. and *Dickeya* spp.

Robert Czajkowski *

University of Gdansk, Intercollegiate Faculty of Biotechnology, University of Gdansk and Medical University of Gdansk, Laboratory of Biologically Active Compounds, A. Abrahama 58, 80-307 Gdansk, Poland

* Correspondence:

Robert Czajkowski

Robert.Czajkowski@biotech.ug.edu.pl

Supplementary Table 2. Distinct and shared ORFs present in genomes of prophages: phiD2, phiDda3, phiDpa1, phiDpa2, phiDze4 and phiDze5 constituting AAI Cluster 1. The number of shared ORFs is shown in bold, whereas the number of distinct ORFs is showed in brackets in italic

**Cluster 1**	**phiD2**	**phiDda3**	**phiDpa1**	**phiDpa2**	**phiDze4**	**phiDze5**
**phiD2**	**49** *(0)*	**49** *(4)*	**29** *(15)*	**29** *(14)*	**34** *(23)*	**35** *(21)*
**phiDda3**	**49** *(0)*	**68** *(0)*	**30** *(15)*	**30** *(14)*	**34** *(23)*	**36** *(20)*
**phiDpa1**	**29** *(11)*	**30** *(15)*	**49** *(0)*	**49** *(1)*	**32** *(21)*	**32** *(22)*
**phiDpa2**	**29** *(11)*	**30** *(15)*	**49** *(0)*	**53** *(0)*	**33** *(20)*	**32** *(22)*
**phiDze4**	**34** *(5)*	**34** *(10)*	**32** *(10)*	**33** *(11)*	**65** *(0)*	**60** *(2)*
**phiDze5**	**35** *(6)*	**36** *(10)*	**32** *(12)*	**32** *(12)*	**60** *(3)*	**70** *(0)*
