# Supplementary material for: May the Phage be With You? Prophage-Like Elements in the Genomes of Soft Rot Pectobacteriaceae: Pectobacterium spp. and Dickeya spp
Source: Front Microbiol. 2019 Feb 14;10:138. doi: 10.3389/fmicb.2019.00138 (PMC6385640; doi:10.3389/fmicb.2019.00138)
Supplement: Supplementary file 6 [file Data_Sheet_6.PDF]

frontiers

Supplementary Material

May the phage be with you? Prophage-like elements in the genomes of Soft Rot *Pectobacteriaceae*: *Pectobacterium* spp. and *Dickeya* spp.

Robert Czajkowski *

University of Gdansk, Intercollegiate Faculty of Biotechnology, University of Gdansk and Medical University of Gdansk, Laboratory of Biologically Active Compounds, A. Abrahama 58, 80-307 Gdansk, Poland

∗ Correspondence:

Robert Czajkowski

Robert.Czajkowski@biotech.ug.edu.pl

Supplementary Table 3. Distinct and shared ORFs present in genomes of prophages: phiD3, phiDda1, phiDda6, phiDdd1, phiDdi5, phiDdi6 and phiDze1 constituting AAI Cluster 2. The number of shared ORFs is shown in bold, whereas the number of distinct ORFs is showed in brackets in italic

**Cluster 2**	**phiD3**	**phiDda1**	**phiDda6**	**phiDdd1**	**phiDdi5**	**phiDdi6**	**phiDze1**
**phiD3**	**46** *(0)*	**39** *(2)*	**42** *(4)*	**41** *(14)*	**38** *(32)*	**39** *(16)*	**37** *(2)*
**phiDda1**	**39** *(4)*	**44** *(0)*	**40** *(4)*	**40** *(15)*	**36** *(34)*	**36** *(21)*	**37** *(2)*
**phiDda6**	**42** *(3)*	**40** *(2)*	**48** *(0)*	**42** *(14)*	**38** *(34)*	**37** *(20)*	**37** *(2)*
**phiDdd1**	**41** *(2)*	**40** *(1)*	**42** *(2)*	**60** *(0)*	**50** *(23)*	**50** *(9)*	**36** *(2)*
**phiDdi5**	**38** *(2)*	**36** *(1)*	**38** *(2)*	**50** *(5)*	**95** *(0)*	**66** *(3)*	**34** *(2)*
**phiDdi6**	**39** *(1)*	**36** *(1)*	**37** *(3)*	**50** *(5)*	**66** *(18)*	**70** *(0)*	**35** *(2)*
**phiDze1**	**37** *(5)*	**37** *(3)*	**37** *(5)*	**36** *(17)*	**34** *(35)*	**35** *(20)*	**40** *(0)*
