# Supplementary material for: May the Phage be With You? Prophage-Like Elements in the Genomes of Soft Rot Pectobacteriaceae: Pectobacterium spp. and Dickeya spp
Source: Front Microbiol. 2019 Feb 14;10:138. doi: 10.3389/fmicb.2019.00138 (PMC6385640; doi:10.3389/fmicb.2019.00138)
Supplement: Supplementary file 7 [file Data_Sheet_7.PDF]

frontiers

Supplementary Material

May the phage be with you? Prophage-like elements in the genomes of Soft Rot *Pectobacteriaceae*: *Pectobacterium* spp. and *Dickeya* spp.

Robert Czajkowski *

University of Gdansk, Intercollegiate Faculty of Biotechnology, University of Gdansk and Medical University of Gdansk, Laboratory of Biologically Active Compounds, A. Abrahama 58, 80-307 Gdansk, Poland

* Correspondence:

Robert Czajkowski

Robert.Czajkowski@biotech.ug.edu.pl

Supplementary Table 4. Distinct and shared ORFs present in genomes of prophages: phiD4, phiD5, phiDd2, phiDze2 and phiDze6 constituting AAI Cluster 3. The number of shared ORFs is shown in bold, whereas the number of distinct ORFs is showed in brackets in italic

**Cluster 3**	**phiD4**	**phiD5**	**phiDdd2**	**phiDze2**	**phiDze6**
**phiD4**	**56** *(0)*	**49** *(2)*	**42** *(2)*	**45** *(4)*	**47** *(3)*
**phiD5**	**49** *(1)*	**55** *(0)*	**45** *(2)*	**45** *(4)*	**49** *(3)*
**phiDdd2**	**42** *(4)*	**45** *(4)*	**52** *(0)*	**39** *(6)*	**43** *(5)*
**phiDze2**	**45** *(3)*	**45** *(4)*	**39** *(4)*	**54** *(0)*	**45** *(5)*
**phiDze6**	**47** *(1)*	**49** *(2)*	**43** *(2)*	**45** *(4)*	**60** *(0)*
