# Supplementary material for: May the Phage be With You? Prophage-Like Elements in the Genomes of Soft Rot Pectobacteriaceae: Pectobacterium spp. and Dickeya spp
Source: Front Microbiol. 2019 Feb 14;10:138. doi: 10.3389/fmicb.2019.00138 (PMC6385640; doi:10.3389/fmicb.2019.00138)
Supplement: Supplementary file 8 [file Data_Sheet_8.PDF]

frontiers

Supplementary Material

May the phage be with you? Prophage-like elements in the genomes of Soft Rot *Pectobacteriaceae*: *Pectobacterium* spp. and *Dickeya* spp.

Robert Czajkowski *

University of Gdansk, Intercollegiate Faculty of Biotechnology, University of Gdansk and Medical University of Gdansk, Laboratory of Biologically Active Compounds, A. Abrahama 58, 80-307 Gdansk, Poland

∗ Correspondence:

Robert Czajkowski

Robert.Czajkowski@biotech.ug.edu.pl

Supplementary Table 5. Distinct and shared ORFs present in genomes of prophages: phiDch1, phiDdi3 and phiDdi1 constituting AAI Cluster 4. The number of shared ORFs is shown in bold, whereas the number of distinct ORFs is showed in brackets in italic

**Cluster 4**	**phiDch1**	**phiDdi3**	**phiDdi1**
**phiDch1**	**65** *(0)*	**42** *(18)*	**42** *(18)*
**phiDdi3**	**42** *(7)*	**81** *(0)*	**81** *(0)*
**phiDdi1**	**42** *(7)*	**81** *(0)*	**81** *(0)*
