# Supplementary material for: May the Phage be With You? Prophage-Like Elements in the Genomes of Soft Rot Pectobacteriaceae: Pectobacterium spp. and Dickeya spp
Source: Front Microbiol. 2019 Feb 14;10:138. doi: 10.3389/fmicb.2019.00138 (PMC6385640; doi:10.3389/fmicb.2019.00138)
Supplement: Supplementary file 9 [file Data_Sheet_9.PDF]

Supplementary Material

May the phage be with you? Prophage-like elements in the genomes of Soft Rot *Pectobacteriaceae*: *Pectobacterium* spp. and *Dickeya* spp.

Robert Czajkowski *

University of Gdansk, Intercollegiate Faculty of Biotechnology, University of Gdansk and Medical University of Gdansk, Laboratory of Biologically Active Compounds, A. Abrahama 58, 80-307 Gdansk, Poland

* Correspondence:

Robert Czajkowski

Robert.Czajkowski@biotech.ug.edu.pl

Supplementary Table 6. Distinct and shared ORFs present in genomes of prophages: phiPa1, phiPa2, phiPcc1, phiPa3 and phiPc2 constituting AAI Cluster 5. The number of shared ORFs is shown in bold, whereas the number of distinct ORFs is showed in brackets in italic

**Cluster 5**	**phiPa1**	**phiPa2**	**phiPcc1**	**phiPa3**	**phiPc2**
**phiPa1**	**81** *(0)*	**39** *(1)*	**22** *(14)*	**24** *(11)*	**24** *(12)*
**phiPa2**	**39** *(30)*	**42** *(0)*	**20** *(16)*	**23** *(11)*	**19** *(18)*
**phiPcc1**	**22** *(39)*	**20** *(9)*	**52** *(0)*	**32** *(5)*	**39** *(7)*
**phiPa3**	**24** *(40)*	**23** *(9)*	**32** *(7)*	**40** *(0)*	**33** *(9)*
**phiPc2**	**24** *(37)*	**19** *(10)*	**39** *(4)*	**33** *(4)*	**49** *(0)*
